# Settlement relationships and their morphological homogeneity across time and scale

**DOI:** 10.1038/s41598-023-38338-9

**Published:** 2023-07-12

**Authors:** Yves M. Räth, Adrienne Grêt-Regamey, Chenjing Jiao, Sidi Wu, Maarten J. van Strien

**Affiliations:** 1 Planning of Landscape and Urban Systems PLUS, ETH Zurich, 8093 Zurich, Switzerland; 2 Chair of Cartography, ETH Zurich, 8093 Zurich, Switzerland

**Keywords:** Environmental social sciences, Urban ecology, Complex networks

## Abstract

Homogeneous settlement morphologies negatively impact urban vibrancy, the environment, and emotions. Mainly resulting from the separation of functions such as work and living, homogeneous settlements have often been found around large cities. However, it remains unknown whether this phenomenon occurs in settlements of any size and persisted over time. In this study, we investigated the relationship between the internal structures of settlements and their location within a settlement network at a large spatial scale and a fine resolution, over seven time steps covering 120 years of settlement development. Using building footprints and road geometries from historical maps of the Swiss Plateau in combination with historical travel speeds, we analyzed networks at both the local- (building networks) and the regional-scale (settlement networks). Our findings show that particularly small settlements located near larger settlements exhibit a high degree of morphological homogeneity, and that this pattern persisted since the early twentieth century despite strong changes in mobility. These results suggest that the position of a settlement within a settlement network can have an impact on its morphological homogeneity, which in turn can have consequences for the functionality and livability of the settlement and provides useful insight to the development of settlements.

## Introduction

Cities are becoming more similar in physical form and structure^1^. This trend of morphological homogenization, i.e. the lack of urban morphometric variability, often goes hand in hand with settlement sprawl and can have negative impacts on urban vibrancy^2,3^, local character^4–7^, and the environment^8–13^. It has also been shown that the homogenization of the landscape in peri-urban areas can lead to negative emotional responses^14^, which is a phenomenon that has been observed worldwide^1^. An important factor driving morphological homogenization is the increased accessibility of settlements with motorized individual transport^15,16^. Given the potentially large influence morphological homogenisation of settlements can have, it is important to gain a thorough understanding of the processes that lead to this homogenization.

Morphological homogenization is not equally prevalent in every settlement. Commuting on a large scale, facilitated by better accessibility due to the motorization of the population, the improved road network and the expansion of public transport, contributed to the homogenization of settlements, as people could fulfill their basic needs such as work, shopping and education in different places^17^. This has led to the creation of satellite settlements that are typically either purely residential or industrial and rely on the accessibility to a larger settlement nearby for the remaining functions^18,19^. Due to the strong focus on a single use of such satellite settlements, their urban form tends to be more homogeneous than their larger neighbors that have a wider variety of services or functions^20,21^. However, research on the relationship between a settlement’s morphology, its relative size and its proximity to other settlements has mainly been limited to isolated focus areas around large cities^1,20,22–25^. Whether these relationships can also be found in areas with many medium- and small-sized settlements remains to be determined. Studying a polycentric settlement structure, including multiple settlements of various sizes across a large area, would allow for the examination of not only the impact of large settlements on smaller ones, but also the influence of medium- and small-sized settlements on even smaller ones. Additionally, due to a lack of historical data, most studies only analyze recent homogenization developments^1,26^. Yet, scholars have long known that settlements are strongly interlinked and share functions and services at different spatial scales^27^. This raises the question whether morphological homogenisation is only a new phenomena or one that has been present for many decades? To answer this question, it is important to consider time series analysis to understand whether and how morphological patterns have changed in the past century.

Spatial analysis using networks at different scales can be used as a tool to provide valuable insights into the interdependence of settlements^28,29^. Settlement network analysis at a regional scale recognizes that settlements are not isolated entities but instead are connected with one another^29–34^. A settlement network allows to compare a given settlement to its connected neighbors in terms of their relative size and proximity. By examining the configuration of the settlement network, we can determine whether a settlement is the largest one within a given radius or whether it is smaller in comparison to its neighboring settlements. Research has demonstrated that such connectivity indicators can aid in categorizing settlements with regard to their rural or urban characteristics^35^. In addition, at a local scale, building networks can be used to analyze a settlement’s form and to delimit settlements themselves^36,37^. In summary, networks can be useful for understanding the settlement internal structure as well as the relationships between settlements and the broader context in which they exist. However, two important challenges exist when assessing the relative size of settlements in settlement networks at different points in time. First, in contrast to euclidean distances, travel times have changed substantially over time^38^. Therefore, linking settlements with travel times is a meaningful proxy to measure the connectivity between any two settlements^39^. Yet, such an analysis requires a large amount of historical data, which is usually lacking. Second, a settlement may be large compared to its direct neighbours, but small compared to all settlements in a region. To be able to compare settlement across such scales, it is necessary to develop a network measure that can identify the relative size of settlements, but that is insensitive to the actual size of the settlement.

Settlement morphometrics has gained considerable attention in recent years, especially due to the availability of high-resolution spatial data^40–42^. Various shape-related metrics of building footprints serve as important indicators to evaluate urban form. These metrics encompass the area^43^, elongation^42^, compactness^44^, and the shape index, which evaluates the complexity of a building footprint^45^. In addition, the orientation of building footprints serves as a crucial aspect of urban form^44^. Scholars then measure the median, or their distribution to determine the homogeneity of settlements^44^. By applying building networks, these metrics can be utilized to compare the morphometric differences of pairs of buildings, thereby taking into account the variations within a settlement rather than focusing solely on aggregated values. Lastly, the distribution patterns of building footprints are also an important component of urban form, which can be quantified with metrics such as the fractal dimension or nearest neighbor index. The fractal dimension^19,46^ measures the complexity and self-similarity of building footprint arrangements in cities, while the nearest neighbor index (NNI)^47^ quantifies the degree of spatial clustering or dispersion of footprints within a settlement. There is thus a large variety of settlement morphometrics that could be used to determine a settlements homogeneity across time and scale.

The aim of this study is to understand how morphological homogeneity is related to a settlement’s position within a road transport network across scales and time. In this study, we define the homogeneity within a settlement’s form by analyzing the variability of differences in morphometrics between pairs of building footprints. Specifically, a settlement is considered more homogeneous when it exhibits a low variability as well as a low median in the differences between connected building footprints. The morphometrics we investigate include, among others, building footprints size, shape, angle as well as spatial distribution metrics. Our research focuses on the Swiss Plateau due to its diverse range of settlements and the availability of historical travel times as well as a unique time-series of historical, topographic maps dating back to 1870, which have now been vectorized for the first time^48^. In our analysis, we consider settlements of a broad range of sizes, including hamlets, villages, towns, and cities. This unique dataset, based on historical building footprints and roads, allows us therefore to examine the form of even the most remote settlements. Additionally, due to the polycentric settlement structure of the Swiss Plateau, it is well-suited for measuring the relationship between relative settlement size and the homogeneity of settlement form. The findings of this research have the potential to inform policies and strategies for addressing the negative impacts of settlement sprawl and homogenization.

## Methods

**Figure 1:**
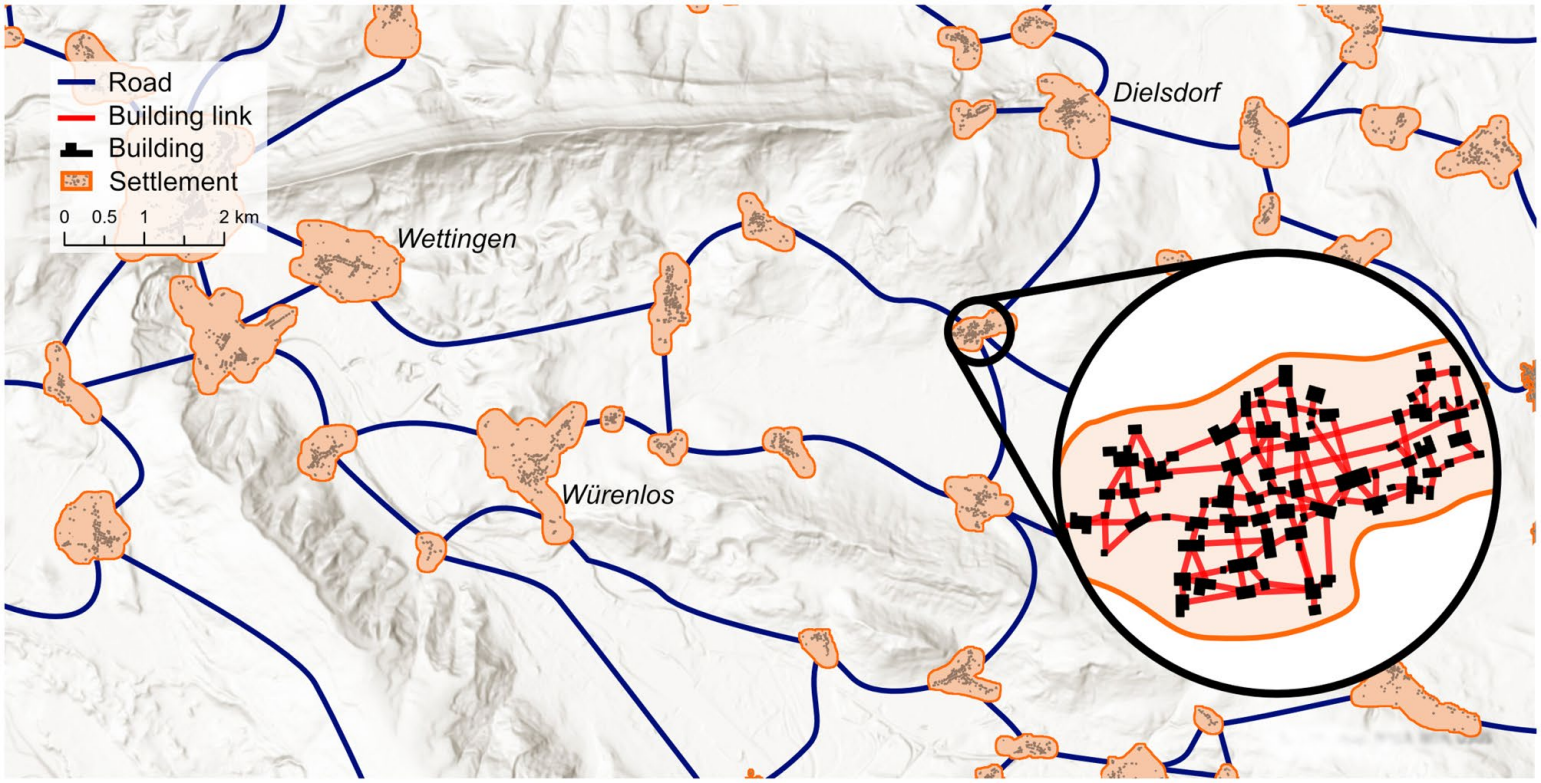
Conceptual overview of the settlement- and building-network. The building network consists of multiple isolated subgraphs, each of which contains at least 10 buildings that are no more than 50 m apart from each other. Each subgraph represents a settlement (i.e. node) in the settlement network. The settlement network connects settlements based on the travel time calculated using the road infrastructure and the travel speed associated with it. Both the building and settlement networks were recalculated for each time step. Hill-shade map source: ESRI, CGIAR, and USGS^76^.

To analyze the relation between intra- and inter-settlement patterns, we applied networks at two different scales: building networks (local scale) and settlement networks (regional scale). Their generation is explained in detail below. Figure 1 provides a conceptual overview of the basis of the two networks and the different scales. We generated these networks for multiple time steps covering the time period from 1899 to 2020, to investigate the consistencies of the relationships between the two scales over time. For the analysis of the the morphological homogeneity of settlements, we applied established morphometrics related to size, shape, orientation and spatial distribution to building networks, focusing on the variability of their differences. Furthermore, to distinguish a settlements size in relation to its neighboring settlements we developed an indicator to assess the spatial dominance using the travel times and settlement sizes as input variables. The source code to perform all analyses has been made available online (see “Data availability” section). For the spatial data preparation, we used primarily the arcpy^49^ library in Python, as well as some helper functions of other libraries^50–54^. For the statistical analysis we used R^55^, where most work was done using functions from the igraph-^56^, sf-^57^, sp-^58^, and the shp2graph-^59^ libraries, while using helper functions from a number of additional libraries^60–71^. The statistical figures were generated using the ggplot2-^72^ and the ggforce-^73^ library. The maps were created with the help of ArcGIS Pro^74^. The conceptual illustrations were generated using Affinity Designer^75^.

### Study area

**Figure 2:**
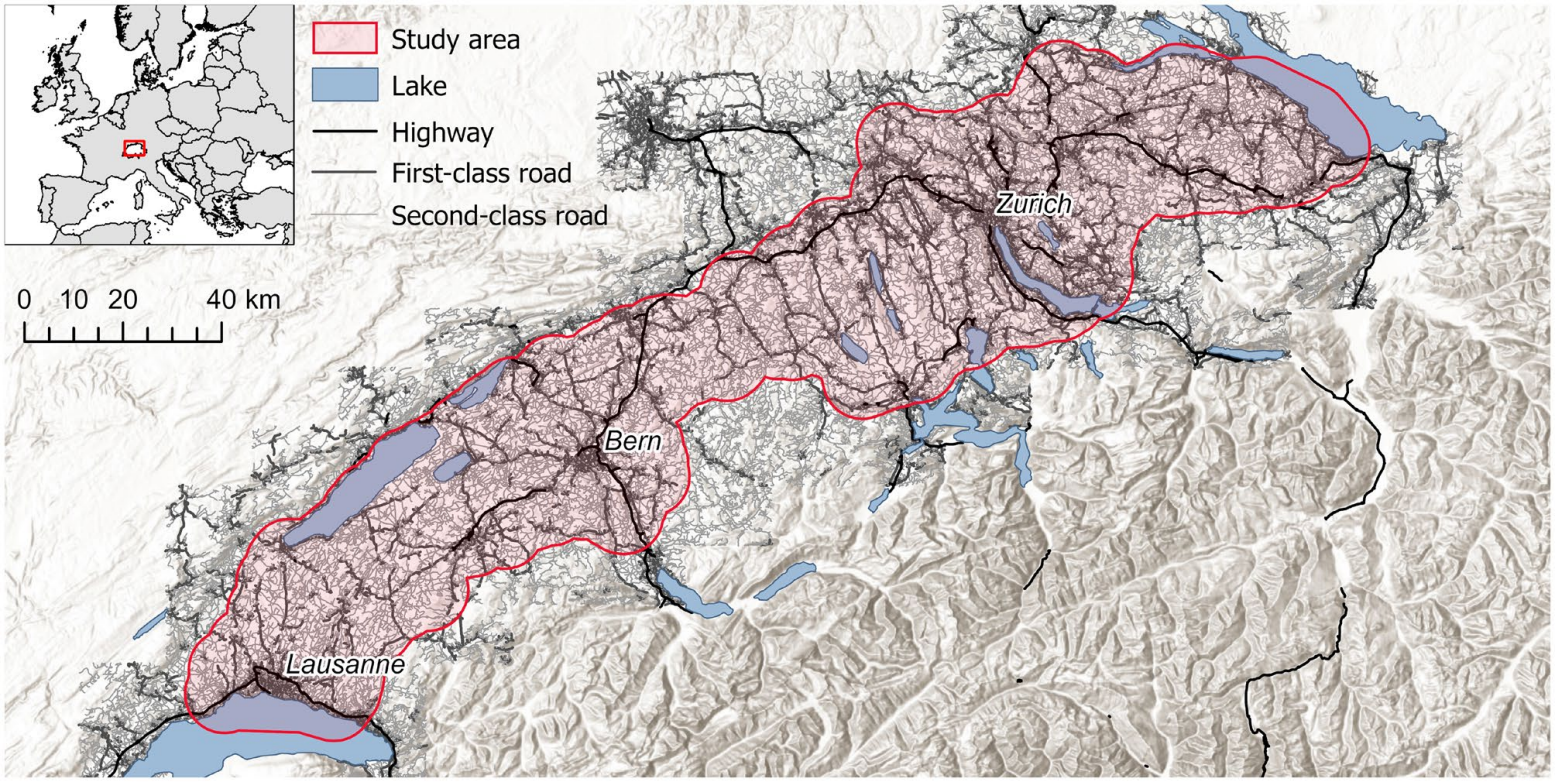
Study area and the used road network extent for 1978. The road network in the study area, which covers most of the Swiss Plateau, is divided into three categories: first-class, second-class, and highways. These categories are used to classify the different travel speeds within the study area. Hill-shade map source: ESRI, CGIAR, and USGS^76^.

The analyses were carried out in the biogeographical region of the Swiss Plateau (approx. 10,000 km$^{2}$)^77^, which is a suitable area to study the development of settlement networks for several reasons. The region consists mainly of a polycentric settlement structure^78^, intermixed with agriculture, forest and water bodies. The region is naturally demarcated by the Jura mountains in the north-west, Lake Constance in the north-east and the Alps in the south. With two thirds of the Swiss population living on the Swiss Plateau, the region is one of the most densely populated regions of Europe (450 people per km$^{2}$) and contains the five largest Swiss cities of Zurich, Geneva, Basel, Lausanne and Bern^79^. Furthermore, a time series of high quality historical maps is available for the entire region (see in “Historical data” section). The cities of Geneva (south-west) and Basel (north) are both connected through rather narrow corridors to the main part of the Swiss Plateau^80^ and are on the border with France and/or Germany. As we do not have the necessary data at the same quality and resolution for these latter two countries, it was, therefore, decided to exclude these cities and their surroundings to prevent bias due to edge effects. We achieve the exclusion of these areas by applying a negative buffer of 7 km (to eliminate the narrow corridors) and expand the result by a positive buffer of 8 km. Figure 2 provides an overview of the resulting study area.

### Historical data

To construct the building and settlement networks for different time steps (1899, 1918, 1933, 1959, 1970, 1978, 2020), we relied on historical maps from which we extracted building footprints and roads. There exists a Swiss-wide topographic map series (so-called *Siegfried Maps*^81^ and *Old National Maps*^82^), which are unique in their spatial- ($< 12$ m, i.e. geometries of building footprints are distinguishable) and temporal-resolution starting in the 1870s^83^. To cover the entire area of the Swiss Plateau, individual sheets from different years had to be combined. This way a non-overlapping time-series covering the entire Swiss Plateau could be generated for the years 1899, 1933, 1959, 1970, 1978, except for the time step for 1918 for which a few map sheets from before 1899 had to be included to achieve complete coverage of the study area. As the sheets were created and updated in no apparent order, there is variation in the time intervals between time-steps (the distribution of the used sheets can be found in Supplementary material Fig. S1). The roads that were extracted from the Siegfried and old national maps were augmented with a time series of highways (at a temporal resolution of 10 years)^29^, after the construction of the first highway in Switzerland in the year 1955. For the time steps 1959, 1970 and 1978, we used the highway networks from, respectively, 1960, 1970 and 1980^29^. The roads and buildings for the time step of 2020 were taken from swissTLM3D, which is a 3D vector dataset (including building footprints, roads, etc.) covering the entirety of Switzerland^84^. Although the Siegfried Maps do depict railway lines (without providing details on their operational status or capacity), and research has investigated the development of public transportation connectivity in Switzerland^38,85^, the necessary temporal and spatial resolution to include rail- and road-based public transportation into our analysis is currently unavailable at a level comparable to that of individual motorized road transport, as this would require historical time tables and exact locations of historical bus stops. Therefore, this work concentrates on car/carriage based road networks.

The building footprints were segmented and vectorized from the historical map sheets using a deep learning algorithm^86^. We adapted an algorithm developed by Heitzler et al.^48^, which was further improved to incorporate mulit-scale contextual information to better distinguish features with similar symbolization. Similar to the building footprints, the road networks were also segmented using U-Net, preceded by a color clustering to extract only the black colored features, such as roads^87–89^. In both cases, the ratio of positive samples (containing objects of interest) vs negative ones are empirically controlled to take into account the sparsity of foreground objects. From the resulting binary rasters, the remaining artifacts (i.e. minor extraction errors) were removed and the features were vectorized using ArcGIS Pro tools^90^. Building footprints have been related with urban social functions^91^, making them a practical and relatively easily obtainable input data for analyzing settlement homogeneity with historical maps. Although other factors, such as building volume, function, or land use, could offer additional insights into settlement homogeneity, such historical data is currently not available at the required resolution in our study area.

In order to calculate carriage and car travel times between settlements, we categorised the extracted roads into three travel speed and capacity classes: highways, first-class roads (higher capacity and generally slightly higher travel speeds), and second-class roads (lower capacities). This differentiation was implemented to enable a more accurate estimation of travel times, taking into account infrastructure changes over time. Additionally, the three categories of car/carriage-accessible roads are distinctly represented in all map products utilized for this study. Although incorporating more road categories, such as forest tracks, might be possible, it is uncertain from the maps whether such roads are car/carriage-accessible, and historical travel speed data for these road types is unavailable. The Siegfried maps distinguish between first-class roads (wider than 5 m and a slope of less than 10%) and second-class roads (3–5 m wide and a slope of less than 15%)^92^. From the old national maps, we also extracted first-class (wider than 6 m) and second-class (wider than 4 m) roads^93^. For the time step of 2020, the swissTLM3D was used from which we also used roads wider than 6 m for the first class and wider than 3 m for the second class of roads^84^. Transport modes like cycling and walking were excluded under the assumption that their accessibility has remained consistent at a regional level, meaning that walking distances have stayed constant and cycling (aside from the recent introduction of e-bikes) has also remained largely unchanged. As previously stated, railways and other forms of public transport were not considered due to insufficient data.

Finally, we assigned travel speeds to each road segment. We generalize travel times for the entire study area, but differentiate by road category and by year. The historic development of the travel speeds on different types of roads has been already extensively studied^38,85,94^. The travel speeds are an approximation of the annual average daily traffic travel speeds for the various roadway types. For time steps prior to 1959, the travel speeds have already been estimated for Switzerland since 1850^85^, and for the time steps between 1959 and 1978, speeds were taken from Erath and Fröhlich^94^. For the time step of 2020 we could use the information from the Swiss national passenger transport model (NPVM 2017)^95^. A summary of the selected travel speeds and their respective literature sources can be found in Supplementary material Table S1.

### Building network and indicators for morphological homogeneity

To analyze the settlement internal morphology, we created a building network (BN) for every time step following the methodology developed by Wang and Burghardt^36^. The network serves as the basis for the morphometric analysis, while it also enabled us to define individual settlements that were used as nodes in the settlement networks. The network was established by creating a link in between two buildings if the shortest edge-to-edge distance between their building footprint is no more than 50 m and there is no other building between the two buildings that intersects the link. A link can therefore be understood as the shortest line of sight between two buildings (ignoring building height due to lack of data availability). The methodology of BN was initially developed to distinguish neighborhoods within cities and the authors tested distance thresholds between 10 and 100 m and had the best performance in identifying morphologically homogeneous neighborhoods with 30 m^36^. However, in our analysis, we focus on entire settlements rather than individual neighborhoods. To do this, we needed to determine a suitable threshold for the building network that satisfied two criteria: small enough to compare adjacent buildings (close to 30 m) and large enough to prevent settlements from being fragmented into neighborhoods. This is particularly important to avoid the division of settlements due to wider roads and rivers, which could lead to an unintuitive fragmentation. During our initial examination of data from 1899, we tested thresholds ranging from 10 to 100 m (Supplementary material Table S2). We discovered that at a 50 m threshold, there was a reduction in the rate of decline of the number of settlements. Particularly, at a 10-m threshold, we identified a much higher number of settlements (99,689) compared to a 20-m threshold (61,213 settlements, decrease of 38.6%). The rate of change dropped to 14.4% when moving from a 40 to 50 m threshold and remained stable thereafter. Taking into account the findings from Wang and Burghardt, local knowledge and visual inspection of the resulting settlements we determined that a 50-m threshold was optimal for this analysis.

To evaluate the homogeneity of settlement morphology, we employed a range of standard metrics from the field of urban morphometrics. These metrics are commonly used to quantify the form of building footprints and the spatial distribution of buildings within settlements:

- *Area*^43^ This metric measures the building footprint area (A) size calculated in square meters.
- *Angle*^44^ This metric quantifies the direction/orientation a building is facing and is measured in degrees, with 0$^{\circ }$ representing north and increasing clockwise to 359$^{\circ }$.
- *Elongation*^42^ This metric is the ratio of the longest dimension (length) of a building to its shortest dimension (width), with values ranging from 0 (highly elongated) to 1 (not elongated).
- *Compactness*^44^ This metric measures how closely a building’s shape resembles a perfect circle, with compactness (C) values ranging from 0 (least compact) to 1 (most compact). ${\text{C}} = \frac{4 \pi \cdot {\text{Area}}}{({\text{Perimeter}})^2}$
- *Shape index*^45^ This metric quantifies the complexity of a building’s shape, with higher shape index (SI) indicating more irregular shapes. ${\text{SI}} = \frac{{\text{Perimeter}}}{\sqrt{{\text{Area}}}}$

Using the BN, we were able to determine the difference of these metrics between pairs of buildings. For the angle metric, we first computed for both building footprints their dominant angles ($\alpha _m$ for building *m* or $\alpha _n$ for building *n*) using “Calculate Polygon Main Angle” operation from the ArcGIS cartography toolbox^96^ and then calculated the absolute difference of the angles. The angle resulting difference can be between 0$^{\circ }$ and 360$^{\circ }$. This requires a transformation because an orientation of 0$^{\circ }$ and 180$^{\circ }$ as well as 360$^{\circ }$ refers to parallel buildings. Moreover, 90$^{\circ }$ and 270$^{\circ }$ oriented buildings also correspond to perpendicular buildings with respect to those oriented in 0$^{\circ }$. To calculate the angular difference between the two dominant building orientations, the transformation with Eq. (1) was used, where a parallel pair of buildings has ${\text{Angle diff.}} = 0^{\circ }$ and diagonally oriented pair of buildings has ${\text{Angle diff.}}=45^{\circ }$ (Supplementary material Fig. S2).

1 $$\begin{aligned} {\text{Angle diff.}}= 45-\left|{{\frac{180^{\circ }\cdot \cos ^{-1} \left( |\cos {\left( {\text{rad}}\left( \alpha _m-\alpha _n\right) \right) }|\right) }{\pi }}-45}\right| \end{aligned}$$

After having measured the differences between for the various metrics, we assessed their distribution within the settlements do determine the homogeneity of the metric. As it cannot be assumed that the values of all metrics are normally distributed in every settlement, we employed the robust metrics median and interquartile range (IQR), which have been shown to be suitable for calculating homogeneity within settlements^40^. We furthermore incorporated the median absolute difference (MAD), which tends to be an even more robust measure compared to the IQR^97^.

In addition to the distribution of building pair differences, we also investigated the distribution pattern of building footprints within a settlement, using the following metrics:

- *Fractal dimension*^46^ This metric measures the spatial distribution of buildings by calculating the fractal dimension (D), using the box-counting method. Rasters with different cell (box) sizes (4 m, 8 m, 16 m, 32 m, 64 m, 128 m, 256 m, 512 m, 1024 m) are created and overlapped with a settlement. For every box size $\epsilon$ the number of non-empty boxes $N(\epsilon )$, i.e. raster cells that do not overlap with a building footprint, is counted and a straight line is fitted to the points (logarithm of box size, logarithm of box count) using linear regression. The slope of the fitted line corresponds to the fractal dimension. A lower D value indicates a more homogeneous dispersal of buildings, while larger D values indicate a more complex, heterogeneous pattern. $D = \frac{\log {N(\epsilon )}}{\log {1/\epsilon }}$
- *Nearest neighbor index (NNI)*^47^ This metric compares the observed mean nearest neighbor distance (NND) to the expected mean nearest neighbor distance for a random distribution of *n* building footprints within the same area. A value close to 0 indicates a high clustering, When the NNI is close to 1, it means the observed NND is similar to the expected NND for a random distribution, indicating spatial randomness. If the NNI is greater than 1, the observed NND is larger than the expected NND, suggesting that the points are more spread out or evenly dispersed across the area, which indicates a more homogeneous dispersal of the building footprints. ${\text{NNI}} = \frac{\overline{{\text{NND}}}}{0.5 \cdot \sqrt{\frac{\text{Area}}{n}}}$

**Figure 3:**
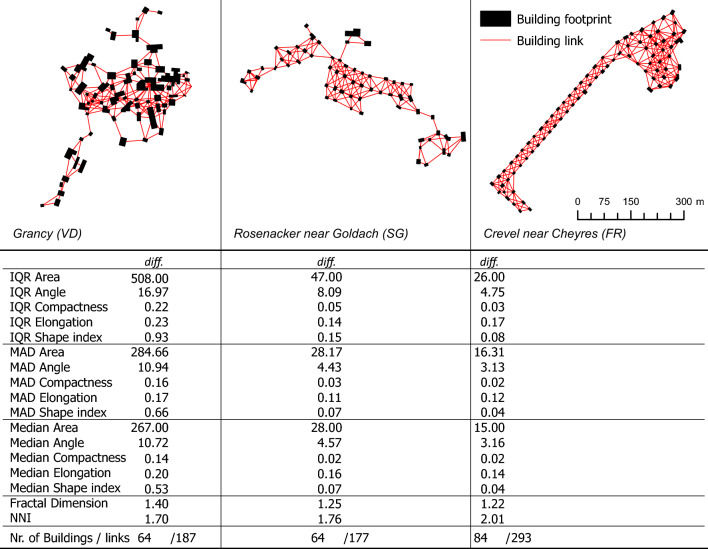
Example settlements from the 1978 time step with increasing homogeneity. All settlements are displayed at the same scale. For visual appeal, the building links are depicted from centroid to centroid.

In Fig. 3 three settlements are presented with all the calculated metrics and it can be clearly seen how the values differ in response to the morphological homogeneity. A low median value for a specific metric implies that there is a minimal disparity or proportion between pairs of buildings, while a low IQR and MAD suggest reduced variability within the settlement. For example, envision a settlement with uniformly sized and spaced building footprints, organized in a circular arrangement, all facing the center akin to the indicators on a watch face. In this situation, the median angle difference would be relatively high as each neighboring building possesses a unique orientation. Nonetheless, the interquartile range (IQR) of the angle difference would equal 0, since every building pair exhibits the same angle difference.

### Settlement networks

After generating the building networks, the settlement networks (SN) were created for each time step. The nodes of the network consist of the settlements and the links are the travel times of the cars or carriages between the settlements. A settlement is defined as a network of buildings.

In our analysis, we considered settlements of varying sizes, including hamlets, villages, towns, and cities. We included hamlets as they often serve as seeds for future settlement growth due to pre-existing infrastructure^98^. We examined multiple thresholds (2–20 buildings) to determine the minimum number of buildings in the BN required for an area to be classified as a settlement (Supplementary material Table S3). While a lower threshold allows for the inclusion of nearly every building as a separate settlement, it may result in noisy data. Conversely, a higher threshold may overlook emerging satellite settlements that are important to include in the analysis. In our initial analysis, we identified a threshold of 10 buildings as the optimal point, above with the rate of decrease of number of settlements becomes notably smaller. For larger thresholds, the rate of decrease of the number of settlements stabilizes at 20% and continues to decline at a slower pace. By using a 10-building threshold, we strike a balance between including too many very small settlements and excluding too many smaller settlements that are potential satellite settlements. Visual inspection of the data across time steps further confirmed that this value is appropriate for capturing important settlements while filtering out extraneous data points. The border of the settlements are defined with a concave hull around the centroids of the building footprints for every isolated building network, using the concaveman function^70^ in R.

Following the geometry extraction of roads and assignment of travel speed, travel time between each pair of settlements was calculated. For this, the settlements were intersected with the road network to create one or several settlement-road intersections per settlement. Each intersection could serve as the origin or destination point for the shortest distance calculation. Between all pairs of settlement-road intersections, we calculated the least-cost path based on the travel time as link weights, using ArcGIS Pro’s “Make Origin Destination Cost Matrix Analysis Layer” function^99^. This calculation provides the shortest travel time between all settlement-road intersections, taking into account the different travel speeds and road categories. From all the possible combinations of settlement-road intersections between two settlements, we selected the shortest travel time for each pair of settlements to represent the settlements’ proximity.

**Figure 4:**
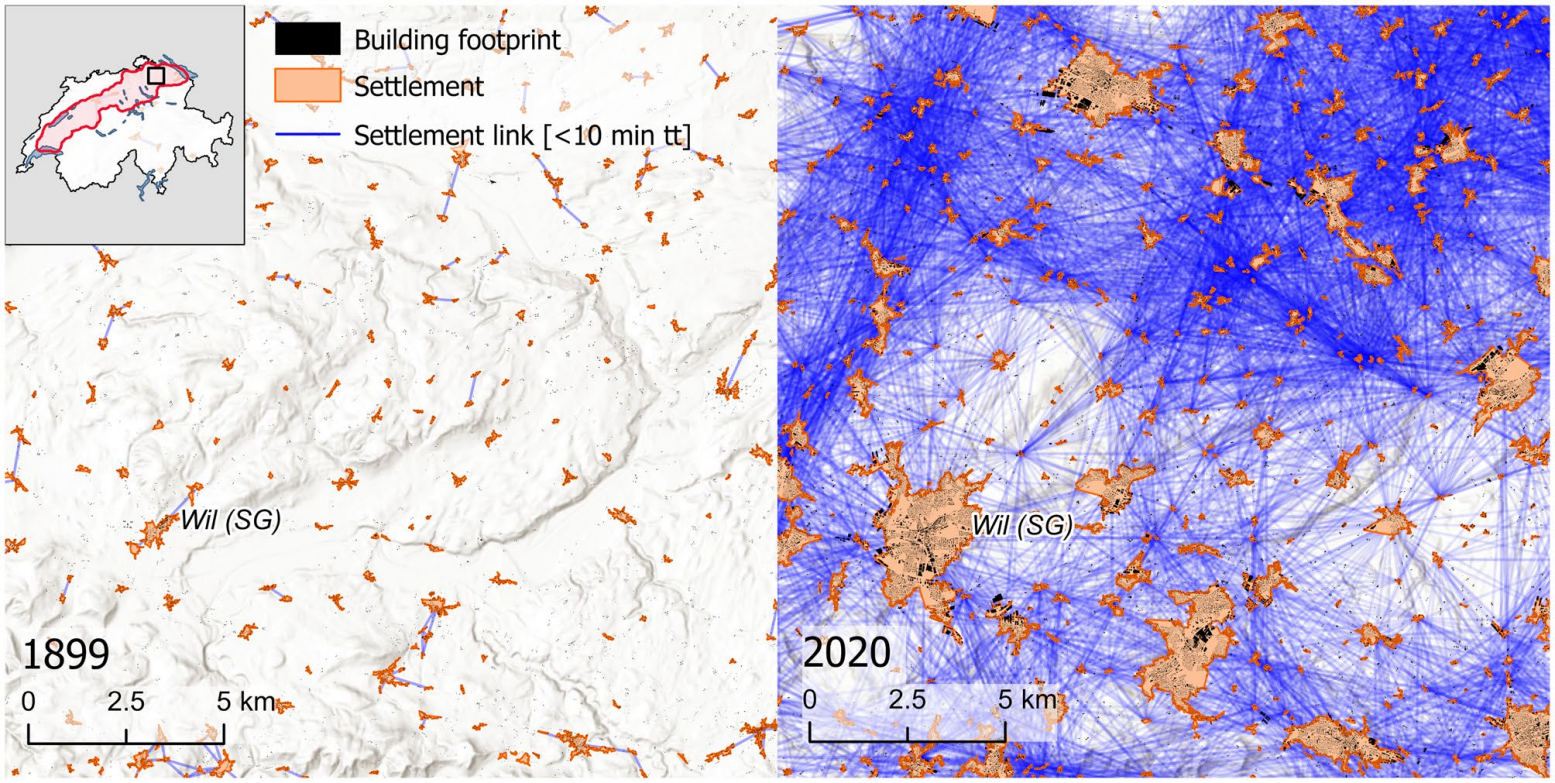
Example of the settlement network evolution between 1899 and 2020. This depiction shows the same area in the north-east of the study area, between Winterthur (ZH) and St. Gallen (SG), at two different time steps. The settlement links represent pairs of settlements that are reachable within 10 min of travel time (tt). The size of the settlements has changed over time, as they have sprawled and merged with one another. Hill-shade map source: ESRI, CGIAR, and USGS^76^.

The result is a settlement network that connects all settlements with one another and each connection has the attribute of the respective travel time. We pruned this network to travel times shorter than than 1 h , as this captures more than 90% of the commutes done by the Swiss residents in 2019^100^. In Fig. 4 two examples are given for settlement networks in 1899 and 2022 for a small section of the study area. For legibility, the depicted networks has been pruned to 10 min travel time (tt) to show the strong change of reach as a result of settlement growth and travel speed increases.

### Distance dependent size ratio

In order to measure the size of a settlement in relation to proximate settlements, we developed a new network indicator $\varphi$, which expresses the ratio of the size of a settlement to the largest settlement in its proximity. For a certain settlement $S_i$, the area of the building footprints is summed to determine the size of a settlement ($\lambda _i$). For a certain maximum travel time *t*, the largest neighboring settlement $S_j$ (with respect to its $\lambda _j$) is selected and its size ratio to $S_i$ is calculated: Eq. (2).

2 $$\begin{aligned} \mu _{it} = \frac{\lambda _i}{\max (\lambda _{jt})} \end{aligned}$$

This means that $\mu _{it}<1$ indicates that a settlement $S_i$ is smaller than its largest neighbor $S_j$ that is reachable within time *t*. Conversely, $\mu _{it}>1$ describes that a settlement $S_i$ is the largest of all reachable settlements $S_j$ within travel time *t*. We calculated $\mu$ for various travel times ranging from 5 to 60 min with 5 min intervals. Thus, each $S_i$ has 12 values of $\mu _{it}$ that either stayed constant or decreased with increasing travel time, since the probability of encountering a larger $\lambda _j$ always increases with travel time. Per settlement, a linear regression was fit between the values of $\mu _{it}$ and the range of travel times. Of interest is the travel time at which the linear regression intersects $\mu _{it}=1$, i.e., the time at which $S_i$ is the same size as its largest neighbor, which we define as $\varphi$ (min). For settlements with smaller settlements in the surrounding, $\varphi$ is greater than 0, while for settlements near larger settlements, $\varphi$ is less than 0.

**Figure 5:**
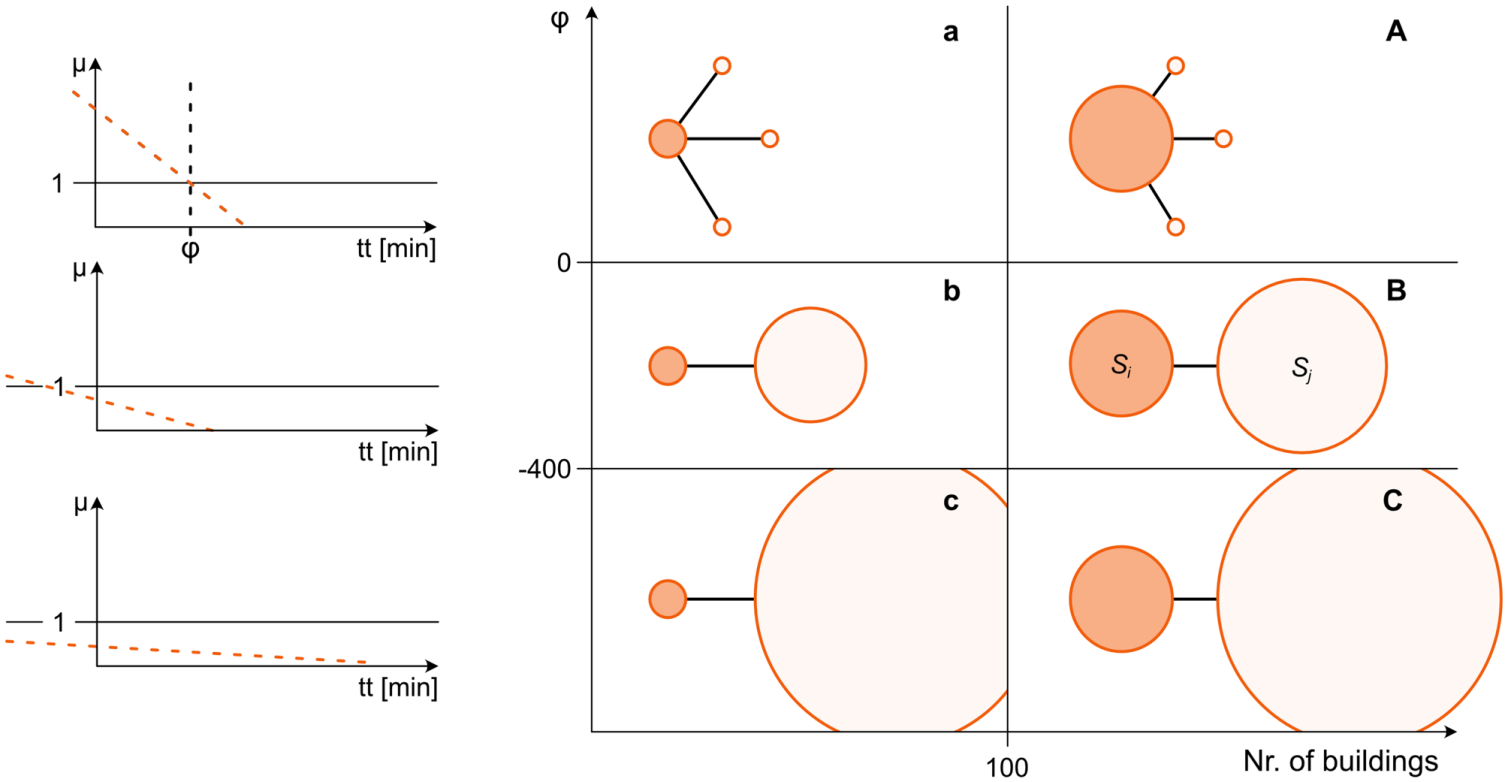
Conceptual overview of settlement relationship types (**a**–**c**, **A**–**C**). On the left are the idealized regression lines to determine $\varphi$ that is necessary to classify the settlement relationships on the right. The circles in the darker shade represent $S_i$ and the lighter shade represent $S_j$ with $\max (\lambda _{jt})$.

This new metric allows us to distinguish settlement relationship types. The advantages of the metric are that it is expressed in travel time that allows the analysis across time steps and that it is scale independent, i.e. very large city surrounded by smaller cities can have the same $\mu$ as a small village surrounded by even smaller hamlets. Making use of this new indicator, we defined six different settlement relationship types, which are conceptually depicted in Fig. 5 (Supplementary material Fig. S3 displays an example for a small region in 1970). First, we distinguished between smaller and larger settlements (x-axis in Fig. 5). As the larger settlements usually consist of several smaller settlements that have grown together, they generally have a more heterogeneous building network. Distinguishing between small and large settlements, therefore, helps to better analyze the underlying patterns characteristic to each of the settlement relationship types. Second, we distinguished between settlements that are larger than their surrounding settlements ($\varphi >0$). For settlements that are smaller than their neighboring settlements, we made a further distinction between those that are near larger settlements ($0>\varphi >=-\,400$) and those that are near very large settlements ($\varphi <-\, 400$; y-axis in Fig. 5). The results are the 6 settlement relationship types (*a*, *b*,* c*, *A*, *B*, *C*) where the capital letter indicates settlements larger than 100 buildings. Relationships with the letter a/A indicate settlements that are larger than those in their surroundings, whereas the b/B and c/C settlements are the four types of settlements smaller than those in their proximate surroundings.

In our final analysis, we examined the relationship between urban morphometrics and different types of settlement relationships, as well as whether there is any pattern that has emerged over the past 120 years. For every settlement relationship type, we tested whether these values were normally distributed using the Shapiro–Wilk test^55^. We utilized a Kruskal–Wallis test for each time step to compare the median values of IQR, MAD, and median morphometrics among various types of settlement relationships. When a significant difference in median was observed, we employed a Bonferroni-corrected Dunn test^101^ to determine which settlement relationship types vary significantly form one-another. As certain morphometrics can be influenced by the number of observations, specifically the number of buildings^102,103^, we carried out a correlation analysis between the settlements’ morphometrics and their number of buildings^55^ . Lastly, we computed statistics on the underlying data (e.g. number of settlements, changes in settlement sizes) and performed a sensitivity analysis on the classification of settlement relationships types.

## Results

The results of our analysis support the hypothesis that a settlement’s morphological homogeneity is related to its position within a settlement network. To further investigate this relationship, we here present results of a few key metrics based on their non-existent or very low correlation with the number of buildings (i.e. correlation coefficients close to 0; ± 0.05; Supplementary material Table S4), and the distinct aspects of the overall pattern they represent (size-, shape-, and angle differences). Nevertheless, the results for the metrics not presented explicitly in this section (including MAD and median) align with the general trends observed in the metrics discussed here. Detailed results for these additional metrics can be found in the Supplementary material Figs. S4 to S6 and Tables S5 to S21. In the analysis of larger settlements, no definitive or consistent link was observed between the settlement relationships type and their morphometrics. Therefore, significance tests on the difference of morphometrics were only carried out on the smaller settlement relationship types *a*, *b*, and *c*. Strong and consistent patterns were most apparent between the smaller settlement relationship types *a*, *b*, and *c*, thus guiding our primary focus to these settlements for significance tests.

**Figure 6:**
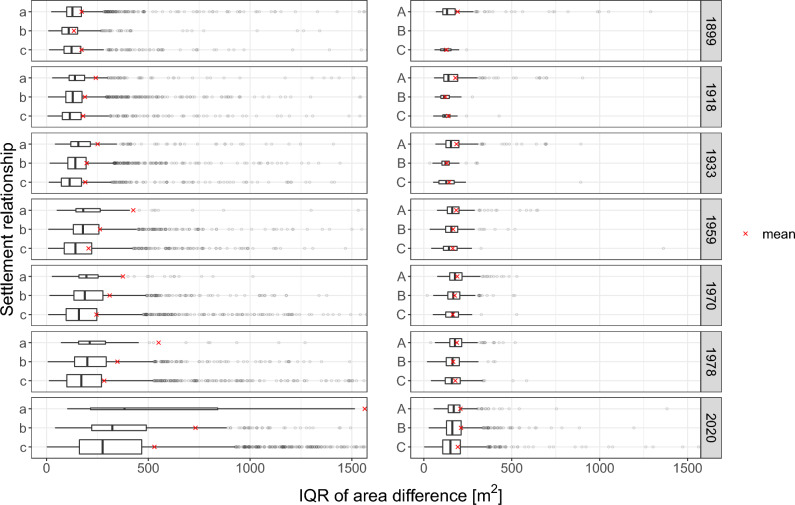
The distribution of the IQR of building pair area differences per settlement relationship and time step. A smaller IQR signifies reduced variability and greater homogeneity within the settlement. The height of the boxplot is directly proportional to the number of observations. A noticeable trend of decreasing values is observed among settlement relationship types *a*, *b*, and *c*. For visualization purposes, the x-axis has been truncated as extreme outliers could potentially obscure the apparent trends.

**Figure 7:**
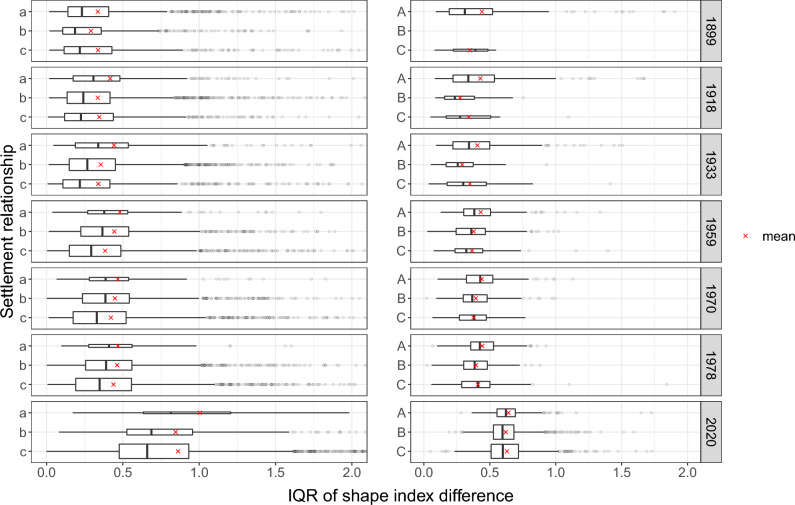
The distribution of the IQR of building pair shape index differences per settlement relationship and time step. A smaller IQR signifies reduced variability and greater homogeneity within the settlement. The height of the boxplot is directly proportional to the number of observations. A noticeable trend of decreasing values is observed among settlement relationship types *a*,* b*, and *c*. For visualization purposes, the x-axis has been truncated as extreme outliers could potentially obscure the apparent trends.

**Figure 8:**
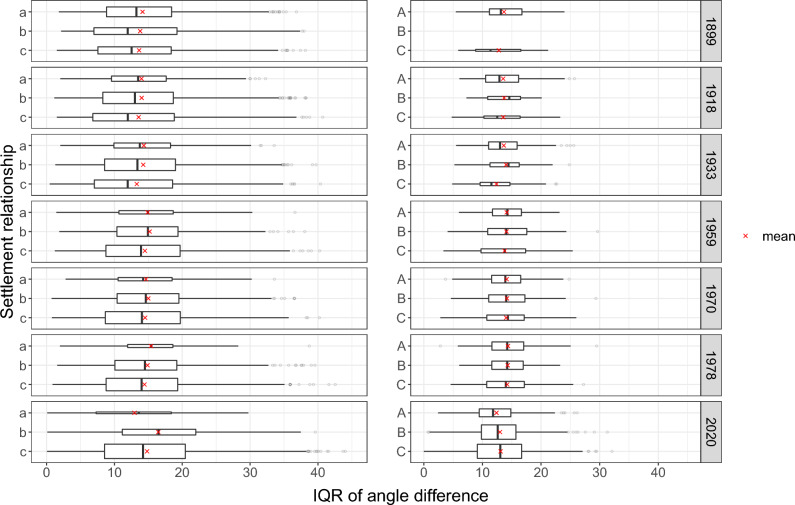
The distribution of the IQR of building pair angle differences per settlement relationship and time step. A smaller IQR signifies reduced variability and greater homogeneity within the settlement. The height of the boxplot is directly proportional to the number of observations. A noticeable trend of decreasing values is observed among settlement relationship types *a*, *b*, and *c*.

For all metrics, we found a consistent trend of increasing homogeneity for, respectively, the small settlement types *a*, *b* and *c* (i.e. settlements below 100 buildings). This was found, among others, for IQR of area differences (Fig. 6), the IQR of the differences in shape index (Fig. 7), as well as the IQR of the pairwise angle difference (Fig. 8). The Bonferroni corrected Dunn’s test indicates that the trend of an increased homogeneity towards *c* is (highly) significant for most metrics and time steps (adj. *p* value: $<0.05$ for most pairings; Supplementary material Tables S5 to S21). For example, in 2020, the median IQR of the area difference (Fig. 6) for *a* is 383m$^{2}$ and significantly higher (adj. *p* value: 0.003) than the 276 m$^{2}$ for *c* (Supplementary material Table S5). Another example to highlight the significant decrease of heterogeneity can be shown with using the shape index (Fig. 7) for 1933 as an instance. For that time step a significant difference is observed in the median IQR of the shape index difference among the pairings (adj. *p* value: $<0.001$). Among the pairings, *a* shows a median IQR difference of 0.338, while for *b* it is 0.268, and 0.218 for *c* (Supplementary material Table S6). For the time step 1978, as yet another example, we find the median IQR angle difference (Fig. 8) to decrease from 15.46 for *a* to 14.50 for *b* to 14.01 for *c* (Supplementary material Table S7). While the angle differences might appear small, they are still significant for the pairings *a*–*c* and *b*–*c* (adj. *p* value: $<0.05$).

The MAD and the median of the pairwise differences mentioned above (area, shape index, angle) exhibit similar trends, where *c* usually displays the greatest level of homogeneity compared to the other two smaller settlement relationship types *a* and *b*. This implies that not only the variability (i.e. IQR and MAD) for these measures is lower, but also their absolute differences are smaller (i.e. median). Moreover, the other building pair metrics related to elongation and compactness differences also adhere to the same pattern, with *c* demonstrating a higher degree of homogeneity in most cases, with high statistical significance (adj. *p* value: $<0.001$). For the larger settlement relationship types, it becomes more difficult to identify a consistent pattern over time. Nevertheless, it can be contended that starting from 1959 large settlements in proximity to larger ones (i.e. *C*) display a relatively low variability in most metrics, and thus high homogeneity, compared to the larger settlements *A*.

The trends described are consistent over time. For the 1899 time step, however, the trends are less clear. For most metrics and time steps, *a* seems to have the most heterogeneous pattern compared to the other smaller settlements, but settlements of type *b* tend to be even more homogeneous compared to settlements of type *c*. Another irregularity can be found in the time step 2020, which often shows a higher variability in the distribution of IQR values per settlement relationship type. Such is the case for the IQR of the differences in the shape index (Fig. 7). The increased variability can be ascribed to the substantially enhanced resolution of the swissTLM3D vector dataset compared to the vectorized historical map sheets. As a result, building footprints display increased complexity. Despite these factors, the pattern of reduced variability in type *c* continues to persist for 2020.

The spatial distribution metrics (NNI and fractal dimension) also show a decreasing homogeneity for the relationship types *a*, *b* and *c* respectively (Supplementary material Fig. S6, Tables S20–S21). However, as these metrics exhibit a weak to moderate positive correlation to the number of buildings in a settlement (Pearson's *r* for fractal dimension: 0.48 and NNI: 0.24), these results need to be interpreted with caution. As we compare settlements of varying sizes, this (weak) correlation can have an influence on the results. Nevertheless, the results do show that type *c* settlements tend to have a lower fractal dimension, which implies less complexity and greater homogeneity in the dispersal of building footprints within a settlement. Conversely, type *c* settlements tend to also have a higher NNI, which suggests a greater regularity in the dispersal of building footprints.

We found that not only the number of settlements varies over time, but also the number of settlements per relationship type. In the results for all three metrics (Figs. 6, 7 and 8), we observe that ever fewer settlements belong to type *a* (1566 in 1899 vs. 50 in 2020), and an increasing number belong to *c* (831 in 1899 vs. 3441 in 2020). This trend can be explained by the fact that with advancing transportation technology and infrastructure, the accessibility of settlements has improved dramatically over the last century^38^. As a result, larger settlements are accessible in a shorter time, decreasing $\mu$ of $S_i$ and increasing the likelihood that $S_i$ is in a settlement relationship *c*. It is surprising that although more and more settlements are in type *c*, either due to the new establishment of satellite settlements or due to the increased accessibility of very large settlements, the median trend between neighborhood types remains mostly consistent. For the larger settlements *A*, *B*, and *C*, this trend is much less pronounced. The overall number of settlements changed from 3346 settlements in 1899 to 6145 by 2020 (Supplementary material Table S22). Also the size of the settlements themselves have increased substantially over time (Supplementary material Fig. S7). The majority of settlements have less than 100 buildings. As expected, the distribution of the settlement sizes also differ between the settlement relationship types (Supplementary material Fig. S8).

The sensitivity analysis of our settlement relationship classification showed that our results were not sensitive to changes in the thresholds delimiting relationship types (Nr. of buildings and $\varphi$; Supplementary material Tables S23 to S51). A shift of ±20% of the threshold values was tested on differences of the median IQR distributions, which led to negligible changes in the results. In fact, the increase of the threshold of $\varphi$ of buildings from − 400 to − 320 lowered the *p* value of the pairwise Dunn’s test for several metrics and multiple time steps, thereby increasing the significant difference of the median IQR distribution of the metrics in between the settlement relationship types.

An analysis of our settlement network showed that the road infrastructure and technological improvements lead to an increase in the accessibility of settlements (the change of reachable settlements within 60 min per time step can be found in Supplementary material Fig. S9). The plateauing in the 2020 time step can be explained by the merging of settlements, i.e. previously separate settlements expanded and grew together, resulting in a decrease of the number of settlements. In particular around the metropolitan area of Zurich this development can clearly be observed. In 2020, the building network of Zurich extends so far that it covers an area that originally, in 1899, consisted of 113 separate building networks (i.e. settlements).

## Discussion

Our work on morphological homogenization in settlements presents a new link between a settlement’s internal structure and its position in a settlement network. The data clearly shows that the settlements morphological homogeneity, expressed in the variability in a number of urban morphometrics of proximate buildings, as well as their spatial distribution, varies significantly with the settlement’s relationship type. In particular we were able to show that smaller settlements ($<100$ buildings), have a more homogeneous form if they are in close commuting range to larger settlements in comparison to settlements that are more remote, or to those that are larger than other proximate settlements. We could also show that this pattern has been present for more than a century, despite the massive change of travel speeds over the past 120 years^38^. Our work highlights the impact of a settlement’s position within a settlement network on its morphological homogeneity. This homogeneity harbours a number of potentially negative impacts on various aspects of urban life^2,3,5–7,14^.

We used carriage and car travel-time based networks to determine the relationship types of settlements within a settlement network. Raster analysis or Euclidean networks would not have been able to show the underlying patterns, as these methodologies lack functional connectivity. As the effective proximity of settlements changes strongly over time^38^, a measure of functional connectivity is desirable when analysing time series. With our approach, using the generalized travel speeds for the different road categories and time steps, we had a proxy for the functional connectivity between the settlements. Additionally, the classification scheme for the settlement relationships allows for comparison of multiple time steps as proximity, unlike Euclidean distance, changes over time.

The distance dependent size ratio has shown to be a useful approach, as it is applicable across scales and suitable for the use on a large, heterogeneous study area with multiple cities that are surrounded by towns, towns surrounded by villages, or villages surrounded by hamlets. Similar to the proximity index^104^, we use the size ratio of neighboring settlements as a variable. Whereas the proximity index uses a pre-defined proximity buffer radius, in which the sum of the size ratios is calculated for every settlement, our metric goes further, in that we include the distance more dynamically between a settlement and its largest neighbor at multiple travel time intervals. With the distance dependent size ratios, the existence of settlements strongly differing in size at close proximity is more emphasized in comparison to the proximity index. As the proximity to larger settlement is assumed to be the main driver of homogenization, our metric is more suitable for the study of this phenomenon and allows us to get a better picture of the settlements relationships across scales.

We found that morphological homogeneity was less affected by the settlement relationships in larger settlements ($>100$ buildings). This is surprising, as most studies that have shown homogenisation focus on larger cities and their surrounding satellite settlements^1,6,13,105^. However, we found that especially in relatively small settlements the settlement relationships have a pronounced effect on settlement homogeneity. There are several potential explanations of this discrepancy with existing literature. First, the smaller settlements can be the result of top down planning dedicated to fulfill specific functions such as industrial sites or residential neighborhoods, which tend to have a more homogeneous structure^15,18,20,106^. Second, larger settlements are often the result of the merger of smaller settlements (Fig. 4) through infill development^107^. The combination of different neighborhoods can result in a more heterogeneous internal structure, potentially reflecting a settlement that provides a range of basic services across different areas. Instead of focusing on entire settlements, a focus on neighborhoods within settlements could perhaps be a relevant extension of our approach to study the processed governing homogenization within large settlements.

What we have also seen is that, unlike the other time steps, the time step of 1899 did not have an apparent relationship between homogeneity of the settlement form and settlement relationship types for most of the applied morphometrics. The lack of a trend for the year 1899 can be explained by the large differences in travel speeds and the resulting accessibility. In Fig. 4 we have shown how disconnected the settlements were in the earliest time step in comparison to the most recent one. This leads to considerably fewer data points for the calculation of the linear regression to estimate $\varphi$. In these cases, the calculation of $\varphi$ is likely less robust and relationships to other variables should be interpreted with caution. At the beginning of the 20th century, satellite settlements were less common due to high travel costs and the need for services to be nearby. Suburban areas, a result of car-oriented urban development^15,108^, were not yet prevalent. Similarly, industrial sites were present but not as significant as those developed later in the 20th century. Nevertheless, we found that the homogenization of settlements is not a recent phenomenon, but can be seen as early as the 20th century. However, with the current aggregated analysis we cannot determine whether homogeneity of certain settlements changed or stayed constant over time. For future research, it would thus be interesting to examine the changes in individual settlements over time and identify the factors that drive their potential homogenization, in addition to the proximity to larger settlements. Since our definition of a settlement is not based on administrative boundaries, such an analysis would present new challenges in tracking expanding and merging settlements over time. This type of analysis is likely to provide further valuable insights in drivers of settlement development.

We opted to utilize well-established morphometrics to examine various aspects of the building footprint geometries, including their shape, orientation, and spatial distribution. By employing the building network, we were able to study the differences among buildings for each morphometric. We then used the median difference, as well as the distribution of those differences (expressed as IQR and MAD), to assess their settlement internal variability, i.e. homogeneity. Intriguingly, the aggregated patterns reveal comparable trends for all the metrics across the majority of time steps. This suggests that the buildings within small settlements surrounding by larger settlements are indeed quite alike in terms of their form, orientation, and spatial distribution, which one would intuitively consider a homogeneous settlement. The use of building networks for morphological analysis is a rather new approach^37^, yet due to its possibility to analyze the relation of proximate buildings at different scales it has proven to be a useful tool for this analysis. Settlement homogenization could also be studied with other indicators, such as landscape metrics^1,23^, economic characteristics or the level of available infrastructure and services. However, in a historical context, such indicators are impractical due to a lack of data or appropriate models. Overall, a holistic approach that incorporates multiple perspectives and indicators is likely to provide a more comprehensive understanding of settlement homogenization.

The travel times we used are based on road-based private transportation. It could be interesting to augment our analysis with a public transport network, as it too is a heavy driver of urbanization processes^15^. However, as the the commuting trip mode share for railway-based public transport has remained below 20% for Switzerland^100^, public transport is still a less relevant proxy for functional connectivity of settlements than road-based private transportation. Furthermore, we expect that every settlement that is well connected via public transport is most likely comparably well-connected for individual transport modes, particularly in the infrastructure dense area of the Swiss Plateau. As our results showed, the individual road transport based commuter network was sufficient to uncover the homogenization patterns relative to the settlements position within the network.

In conclusion, the research presented in this paper has shown that the morphological homogeneity of settlements is influenced by their position within a settlement network, with smaller settlements having more homogeneous urban form when they are in close commuting range to larger settlements. This finding has potential implications for understanding the impacts of urbanization on various aspects of urban life, as well as for modeling settlement development. The use of network analysis and a settlement-size independent measure for classifying settlement relationship types in this study allowed for an analysis of settlement morphology and its relation to settlement networks across time and across scale.

## Supplementary Information

Supplementary Information.

## Data Availability

The source code for generating and analyzing the data can be found at the following URL: https://github.com/yraeth/bnet-snet. The Data will be available upon reasonable request from the corresponding author.
