# Supplementary material for: Settlement relationships and their morphological homogeneity across time and scale
Source: Sci Rep. 2023 Jul 12;13:11248. doi: 10.1038/s41598-023-38338-9 (PMC10338443; doi:10.1038/s41598-023-38338-9)
Supplement: Supplementary file 1 — Supplementary Information. [file 41598_2023_38338_MOESM1_ESM.pdf]

Settlement relationships and their morphological homogeneity across time and scale

Yves Marc Räth[1,*], Adrienne Grêt-Regamey[1], Chenjing Jiao[2], Sidi Wu[2], and Maarten J. van Strien[1]

[1]ETH Zurich, Planning of Landscape and Urban Systems PLUS, Zurich, 8093, Switzerland
[2]ETH Zurich, Chair of Cartography, Zurich, 8093, Switzerland
[*]yraeth@ethz.ch

Supplementary Material

Number of Sheets per time step

In Fig. S1 the distribution of the sheets per time step is shown. Due to the increasing sheet size after the Siegfried maps, fewer sheets were necessary to complete the coverage of the study area.

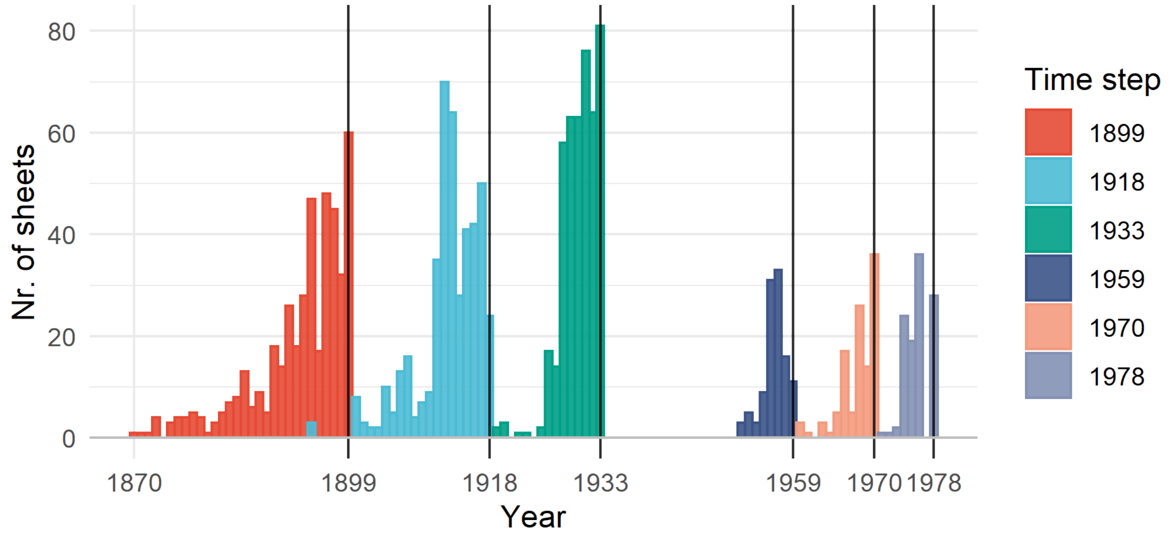

Figure S1. Map sheets per time step. The map sheets drawn before 1949 each cover an area of 52.2 km^2 [1], while the later ones cover an area of 210 km^2 [2].

Used travel speeds

The travel times in Tab. S1 show how the average speed has increased over the years, plateaued in the 70's and reduced since. This is due to increasing congestion, environmental policies and reducing of travel speeds for safety reasons. We ignored the effect of elevation on travel speed, as the same amount of traffic can be expected in both directions, i.e., the increase in downhill travel speed is cancelled out by the decrease in uphill travel speed on the exact same road. Furthermore, we did not take into account specific road capacities, congestion for trips through settlements, or penalties for intersections or road changes.

Table S1. Travel speeds by road class and year. For all years, we treated the roads as if they had an elevation of <4%, and the year closest to the time steps used in this study was always chosen.

	travel speed [km/h]		
time step	second-class	first-class	highway
1899[3]	3	4	
1918[3]	20	30	
1933[3]	20	30	
1959[4]	60	83	90
1970[4]	84	86	112
1978[4]	85	87	118
2020[5]	44	55	97

Number of settlements relative to building distnace threshold

Table S2. Building network distance threshold vs. number of settlements and rate of decrease. Based on the rate of decrease, the insights from Wang and Burghardt, as well as visual inspections lead to the choice of 50 meters as the most suitable threshold.

Threshold [m]	Nr. of Settlements	Rate of decrease [%]
10	99,689	
20	61,213	38.6
30	48,390	20.9
40	40,816	15.6
50	34,951	14.4
60	29,939	14.3
70	25,653	14.3
80	22,062	14.0
90	18,829	14.7
100	16,038	14.8

Example of building angle differences

In Fig. S2 extreme cases of the building angle differences are shown.

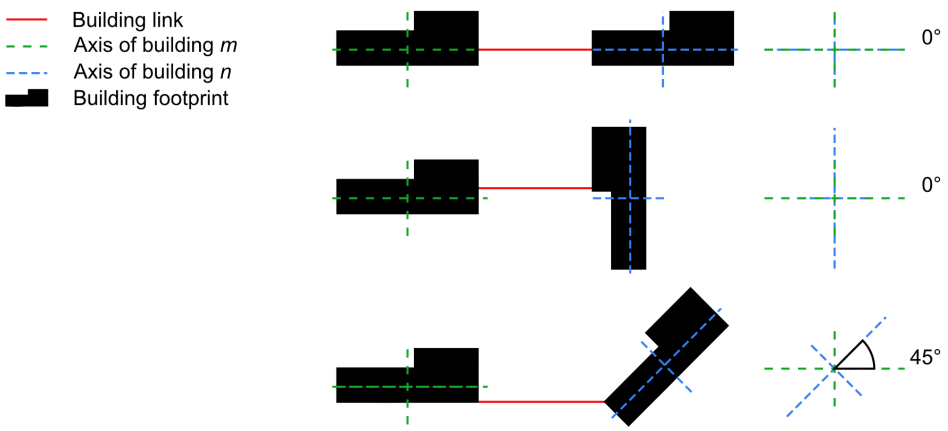

Figure S2. Overview of the angle similarity of building pairs. The upper two building pairs are orthogonal to one-another resulting in a 0° angle difference. The lower pair has a 45° angle difference, the highest value possible.

Number of settlements relative to Nr. of buildings threshold

Table S3. Number of buildings threshold vs. number of settlements and rate of decrease. Based on the rate of decrease and the visual inspections the threshold of 10 building was seen as most suitable.

Nr. of Buildings	Nr. of Settlements	Rate of decrease [%]
2	20,083	
4	9,948	50.5
6	6,386	35.8
8	4,643	27.3
10	3,714	20.0
12	3,117	16.1
14	2,732	12.4
16	2,440	10.7
18	2,213	9.3
20	2,032	8.2

Example of settlement relationship types

In Fig. S3 an example is shown on how the settlement relationship types are distributed in space.

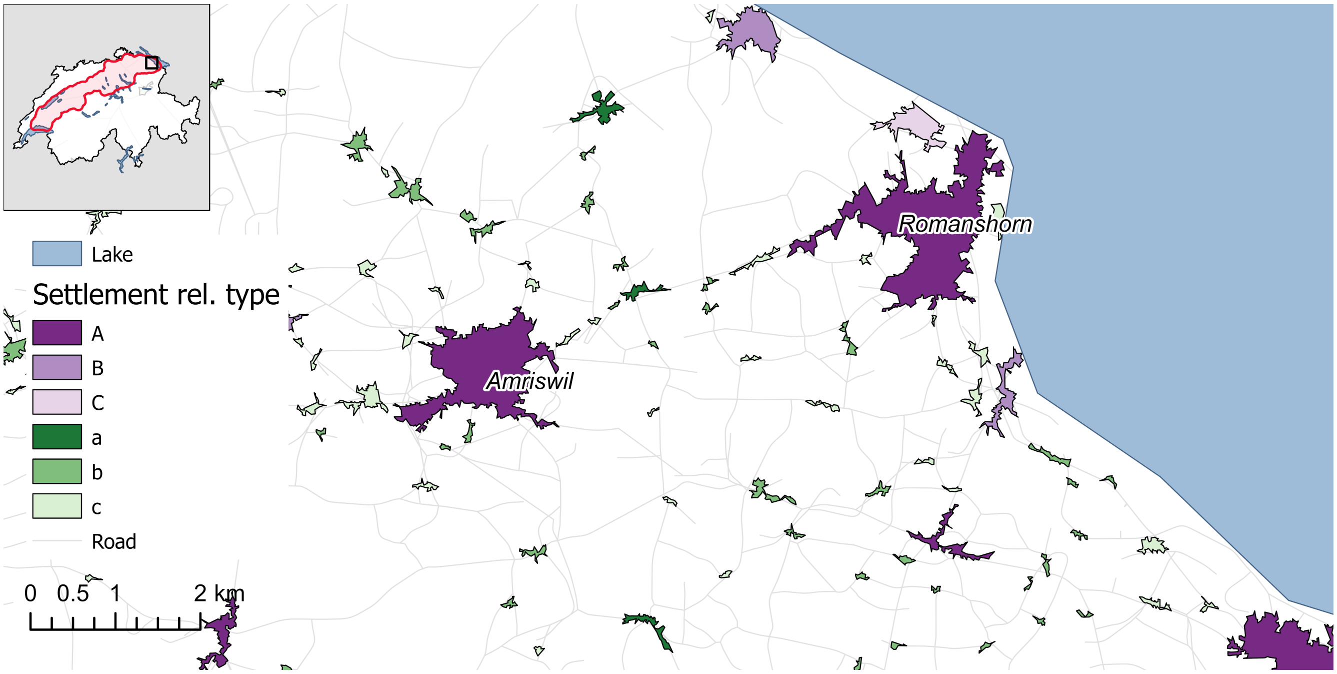

Figure S3. Example of the settlement relationship types in the north-eastern region of the study area for the time step of 1970.

Correlation between morphometrics and number of buildings per settlement

Table S4. The Pearson correlation coefficient *r* between urban morphometric characteristics and the number of buildings per time step. In the final column, the absolute average correlation between the two variables is displayed.

		1899	1918	1933	1959	1970	1978	2020	mean
IQR	Area diff.	0.02	-0.01	-0.02	-0.04	-0.04	-0.04	-0.03	0.03
	Angle diff.	-0.02	-0.03	-0.02	-0.04	-0.03	-0.03	-0.07	0.03
	Compactness diff.	0.08	0.07	0.04	0.00	-0.01	-0.00	-0.05	0.04
	Elongation diff.	0.05	0.04	0.04	0.07	0.05	0.05	-0.03	0.05
	Shape index diff.	0.07	0.05	0.02	-0.02	-0.03	-0.04	-0.03	0.04
MAD	Area diff.	0.01	-0.01	-0.04	-0.06	-0.05	-0.08	-0.05	0.04
	Angle diff.	-0.03	-0.05	-0.05	-0.07	-0.07	-0.07	-0.10	0.06
	Compactness diff.	0.07	0.04	-0.00	-0.04	-0.06	-0.07	-0.06	0.05
	Elongation diff.	0.04	0.03	0.02	0.04	0.03	0.03	-0.04	0.03
	Shape index diff.	0.05	0.04	-0.02	-0.05	-0.07	-0.08	-0.04	0.05
median	Area diff.	-0.03	-0.05	-0.07	-0.10	-0.07	-0.09	-0.04	0.06
	Angle diff.	-0.06	-0.09	-0.09	-0.12	-0.12	-0.12	-0.11	0.10
	Compactness diff.	0.03	-0.01	-0.05	-0.09	-0.11	-0.13	-0.08	0.07
	Elongation diff.	0.01	-0.02	-0.04	-0.05	-0.05	-0.06	-0.06	0.04
	Shape index diff.	0.02	-0.00	-0.05	-0.09	-0.10	-0.13	-0.05	0.06
	Fractal Dimension	0.41	0.52	0.50	0.55	0.51	0.58	0.30	0.48
	NNI	-0.19	-0.25	-0.24	-0.28	-0.25	-0.31	-0.14	0.24

Analysis on the significant differences of the median IQR distribution of the pairwise differences per settlement relationship type

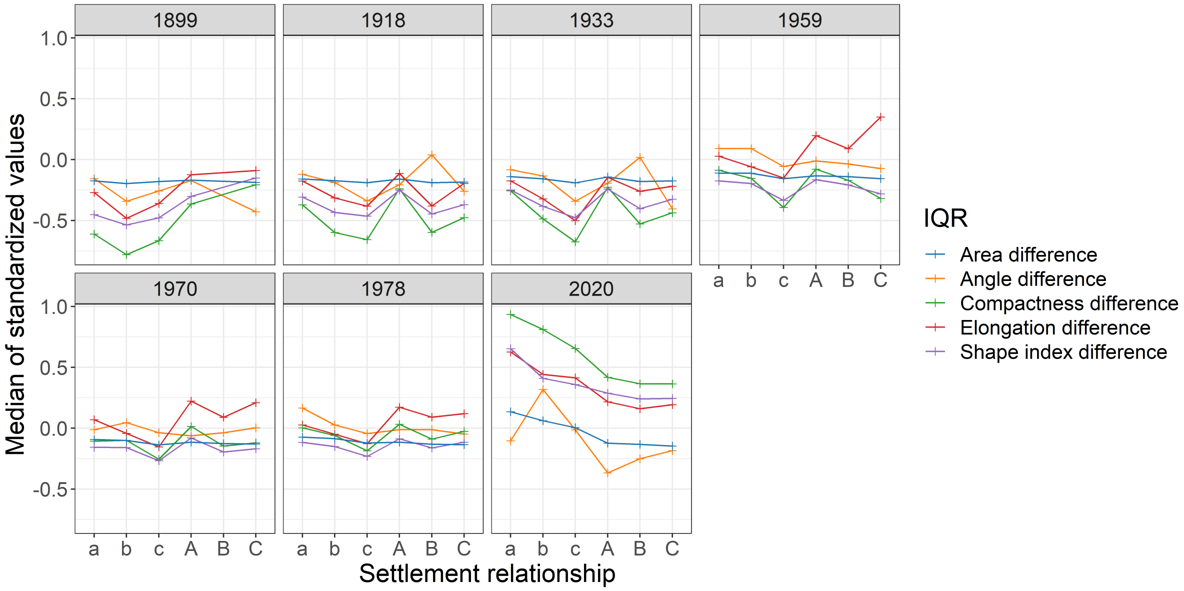

Figure S4. Median IQR distribution of urban morphometric differences per building pair for settlement relationships per time step: Lower median MAD values indicate greater intra-settlement homogeneity. The values have been standardized in order to improve the clarity of the figure and facilitate comparison of trends.

Table S5. Kruskal–Wallis (KW) test and pairwise Dunn's (D) test with Bonferroni correction for p-values on the difference of the IQR pairwise area difference of the settlement relationship types a, b, and c for every time step. The Dunn's test was exclusively utilized for time steps in which the p-value of the KW test was less than 0.05.

Time step	Type 1	Type 2	n 1	n 2	median 1	median 2	KW p-value	adj. D p-value	*sign.*
1899	a	b	1566	744	127	109	<0.001	<0.001	***
1899	a	c	1566	831	127	123	<0.001	0.001	**
1899	b	c	744	831	109	123	<0.001	<0.001	***
1918	a	b	458	2149	140	129	<0.001	<0.001	***
1918	a	c	458	885	140	114	<0.001	<0.001	***
1918	b	c	2149	885	129	114	<0.001	<0.001	***
1933	a	b	414	2233	156	142	<0.001	<0.001	***
1933	a	c	414	1098	156	114	<0.001	<0.001	***
1933	b	c	2233	1098	142	114	<0.001	<0.001	***
1959	a	b	179	1525	180	179	<0.001	0.157	
1959	a	c	179	2068	180	142	<0.001	<0.001	***
1959	b	c	1525	2068	179	142	<0.001	<0.001	***
1970	a	b	176	1496	196	188	<0.001	0.245	
1970	a	c	176	2390	196	159	<0.001	<0.001	***
1970	b	c	1496	2390	188	159	<0.001	<0.001	***
1978	a	b	147	1562	212	201	<0.001	0.18	
1978	a	c	147	2572	212	171	<0.001	<0.001	***
1978	b	c	1562	2572	201	171	<0.001	<0.001	***
2020	a	b	50	538	383	323	<0.001	0.221	
2020	a	c	50	3441	383	276	<0.001	0.003	**
2020	b	c	538	3441	323	276	<0.001	<0.001	***

Sign. levels: *** : p < 0.001, ** : p < 0.01, * : p < 0.05

Table S6. Kruskal–Wallis (KW) test and pairwise Dunn's (D) test with Bonferroni correction for p-values on the difference of the IQR pairwise shape index difference of the settlement relationship types a, b, and c for every time step. The Dunn's test was exclusively utilized for time steps in which the p-value of the KW test was less than 0.05.

Time step	Type 1	Type 2	n 1	n 2	median 1	median 2	KW p-value	adj. D p-value	*sign.*
1899	a	b	1566	744	0.232	0.186	<0.001	<0.001	***
1899	a	c	1566	831	0.232	0.218	<0.001	0.025	*
1899	b	c	744	831	0.186	0.218	<0.001	0.003	**
1918	a	b	458	2149	0.307	0.241	<0.001	<0.001	***
1918	a	c	458	885	0.307	0.226	<0.001	<0.001	***
1918	b	c	2149	885	0.241	0.226	<0.001	0.236	
1933	a	b	414	2233	0.338	0.268	<0.001	<0.001	***
1933	a	c	414	1098	0.338	0.218	<0.001	<0.001	***
1933	b	c	2233	1098	0.268	0.218	<0.001	<0.001	***
1959	a	b	179	1525	0.378	0.369	<0.001	0.331	
1959	a	c	179	2068	0.378	0.300	<0.001	<0.001	***
1959	b	c	1525	2068	0.369	0.300	<0.001	<0.001	***
1970	a	b	176	1496	0.387	0.390	<0.001	0.5	
1970	a	c	176	2390	0.387	0.334	<0.001	<0.001	***
1970	b	c	1496	2390	0.390	0.334	<0.001	<0.001	***
1978	a	b	147	1562	0.409	0.393	<0.001	0.652	
1978	a	c	147	2572	0.409	0.355	<0.001	0.016	*
1978	b	c	1562	2572	0.393	0.355	<0.001	<0.001	***
2020	a	b	50	538	0.815	0.687	<0.001	0.061	
2020	a	c	50	3441	0.815	0.660	<0.001	0.005	**
2020	b	c	538	3441	0.687	0.660	<0.001	0.017	*

Sign. levels: *** : p < 0.001, ** : p < 0.01, * : p < 0.05

Table S7. Kruskal–Wallis (KW) test and pairwise Dunn's (D) test with Bonferroni correction for p-values on the difference of the IQR pairwise angle difference of the settlement relationship types a, b, and c for every time step. The Dunn's test was exclusively utilized for time steps in which the p-value of the KW test was less than 0.05.

Time step	Type 1	Type 2	n 1	n 2	median 1	median 2	KW p-value	adj. D p-value	*sign.*
1899	a	b	1566	744	13.24	11.97	0.004	0.011	*
1899	a	c	1566	831	13.24	12.54	0.004	0.01	*
1899	b	c	744	831	11.97	12.54	0.004	1	
1918	a	b	458	2149	13.49	13.02	0.006	0.371	
1918	a	c	458	885	13.49	11.98	0.006	0.006	**
1918	b	c	2149	885	13.02	11.98	0.006	0.012	*
1933	a	b	414	2233	13.75	13.39	<0.001	0.395	
1933	a	c	414	1098	13.75	11.97	<0.001	<0.001	***
1933	b	c	2233	1098	13.39	11.97	<0.001	<0.001	***
1959	a	b	179	1525	14.95	14.94	0.002	0.996	
1959	a	c	179	2068	14.95	13.93	0.002	0.403	
1959	b	c	1525	2068	14.94	13.93	0.002	<0.001	***
1970	a	b	176	1496	14.24	14.64	0.024	0.775	
1970	a	c	176	2390	14.24	14.06	0.024	0.932	
1970	b	c	1496	2390	14.64	14.06	0.024	0.009	**
1978	a	b	147	1562	15.46	14.50	0.006	0.333	
1978	a	c	147	2572	15.46	14.01	0.006	0.04	*
1978	b	c	1562	2572	14.50	14.01	0.006	0.015	*
2020	a	b	50	538	13.61	16.50	<0.001	0.006	**
2020	a	c	50	3441	13.61	14.22	<0.001	0.269	
2020	b	c	538	3441	16.50	14.22	<0.001	<0.001	***

Sign. levels: *** : $p < 0.001$, ** : $p < 0.01$, * : $p < 0.05$

Table S8. Kruskal–Wallis (KW) test and pairwise Dunn's (D) test with Bonferroni correction for p-values on the difference of the IQR pairwise compactness difference of the settlement relationship types a, b, and c for every time step. The Dunn's test was exclusively utilized for time steps in which the p-value of the KW test was less than 0.05.

Time step	Type 1	Type 2	n 1	n 2	median 1	median 2	KW p-value	adj. D p-value	*sign.*
1899	a	b	1566	744	0.077	0.063	<0.001	<0.001	***
1899	a	c	1566	831	0.077	0.072	<0.001	0.022	*
1899	b	c	744	831	0.063	0.072	<0.001	0.004	**
1918	a	b	458	2149	0.096	0.078	<0.001	<0.001	***
1918	a	c	458	885	0.096	0.073	<0.001	<0.001	***
1918	b	c	2149	885	0.078	0.073	<0.001	0.211	
1933	a	b	414	2233	0.105	0.087	<0.001	<0.001	***
1933	a	c	414	1098	0.105	0.071	<0.001	<0.001	***
1933	b	c	2233	1098	0.087	0.071	<0.001	<0.001	***
1959	a	b	179	1525	0.119	0.113	<0.001	0.204	
1959	a	c	179	2068	0.119	0.094	<0.001	<0.001	***
1959	b	c	1525	2068	0.113	0.094	<0.001	<0.001	***
1970	a	b	176	1496	0.117	0.118	<0.001	0.484	
1970	a	c	176	2390	0.117	0.105	<0.001	0.001	**
1970	b	c	1496	2390	0.118	0.105	<0.001	<0.001	***
1978	a	b	147	1562	0.126	0.121	<0.001	0.481	
1978	a	c	147	2572	0.126	0.111	<0.001	0.008	**
1978	b	c	1562	2572	0.121	0.111	<0.001	<0.001	***
2020	a	b	50	538	0.201	0.191	<0.001	1	
2020	a	c	50	3441	0.201	0.179	<0.001	0.372	
2020	b	c	538	3441	0.191	0.179	<0.001	<0.001	***

Sign. levels: *** : $p < 0.001$, ** : $p < 0.01$, * : $p < 0.05$

Table S9. Kruskal–Wallis (KW) test and pairwise Dunn's (D) test with Bonferroni correction for p-values on the difference of the IQR pairwise elongation difference of the settlement relationship types a, b, and c for every time step. The Dunn's test was exclusively utilized for time steps in which the p-value of the KW test was less than 0.05.

Time step	Type 1	Type 2	n 1	n 2	median 1	median 2	KW p-value	adj. D p-value	*sign.*
1899	a	b	1566	744	0.194	0.181	<0.001	<0.001	***
1899	a	c	1566	831	0.194	0.188	<0.001	0.007	**
1899	b	c	744	831	0.181	0.188	<0.001	0.007	**
1918	a	b	458	2149	0.199	0.191	<0.001	0.003	**
1918	a	c	458	885	0.199	0.187	<0.001	<0.001	***
1918	b	c	2149	885	0.191	0.187	<0.001	0.04	*
1933	a	b	414	2233	0.199	0.190	<0.001	0.003	**
1933	a	c	414	1098	0.199	0.179	<0.001	<0.001	***
1933	b	c	2233	1098	0.190	0.179	<0.001	<0.001	***
1959	a	b	179	1525	0.212	0.207	<0.001	0.15	
1959	a	c	179	2068	0.212	0.201	<0.001	0.002	**
1959	b	c	1525	2068	0.207	0.201	<0.001	<0.001	***
1970	a	b	176	1496	0.214	0.208	<0.001	0.023	*
1970	a	c	176	2390	0.214	0.201	<0.001	<0.001	***
1970	b	c	1496	2390	0.208	0.201	<0.001	0.004	**
1978	a	b	147	1562	0.212	0.207	<0.001	0.164	
1978	a	c	147	2572	0.212	0.202	<0.001	0.004	**
1978	b	c	1562	2572	0.207	0.202	<0.001	<0.001	***
2020	a	b	50	538	0.249	0.237	0.915		
2020	a	c	50	3441	0.249	0.236	0.915		
2020	b	c	538	3441	0.237	0.236	0.915		

Sign. levels: *** : p < 0.001, ** : p < 0.01, * : p < 0.05

Analysis on the significant differences of the median MAD distribution of the pairwise differences per settlement relationship type

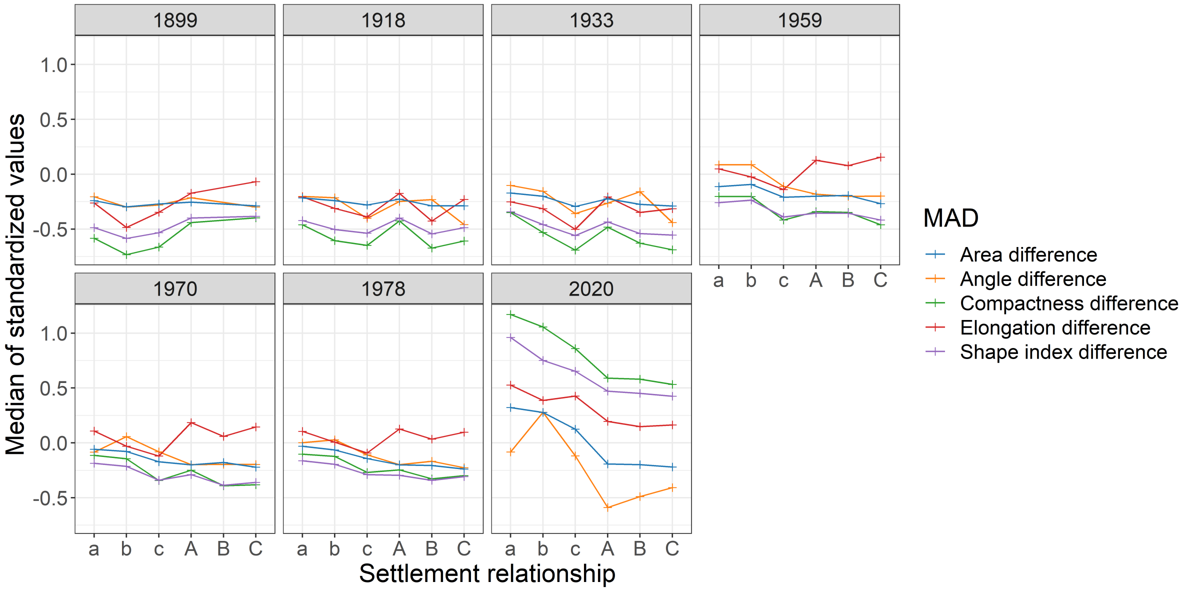

Figure S5. Median MAD distribution of urban morphometric differences per building pair for settlement relationships per time step: Lower median MAD values indicate greater intra-settlement homogeneity. The values have been standardized in order to improve the clarity of the figure and facilitate comparison of trends.

Table S10. Kruskal–Wallis (KW) test and pairwise Dunn's (D) test with Bonferroni correction for p-values on the difference of the MAD pairwise area difference of the settlement relationship types a, b, and c for every time step. The Dunn's test was exclusively utilized for time steps in which the p-value of the KW test was less than 0.05.

Time step	Type 1	Type 2	n 1	n 2	median 1	median 2	KW p-value	adj. D p-value	*sign.*
1899	a	b	1566	744	89	76	<0.001	<0.001	***
1899	a	c	1566	831	89	82	<0.001	<0.001	***
1899	b	c	744	831	76	82	<0.001	0.001	**
1918	a	b	458	2149	95	89	<0.001	<0.001	***
1918	a	c	458	885	95	80	<0.001	<0.001	***
1918	b	c	2149	885	89	80	<0.001	<0.001	***
1933	a	b	414	2233	104	98	<0.001	0.002	**
1933	a	c	414	1098	104	77	<0.001	<0.001	***
1933	b	c	2233	1098	98	77	<0.001	<0.001	***
1959	a	b	179	1525	117	122	<0.001	0.68	
1959	a	c	179	2068	117	96	<0.001	<0.001	***
1959	b	c	1525	2068	122	96	<0.001	<0.001	***
1970	a	b	176	1496	129	125	<0.001	0.346	
1970	a	c	176	2390	129	104	<0.001	<0.001	***
1970	b	c	1496	2390	125	104	<0.001	<0.001	***
1978	a	b	147	1562	135	128	<0.001	0.48	
1978	a	c	147	2572	135	110	<0.001	<0.001	***
1978	b	c	1562	2572	128	110	<0.001	<0.001	***
2020	a	b	50	538	213	203	<0.001	0.401	
2020	a	c	50	3441	213	169	<0.001	0.008	**
2020	b	c	538	3441	203	169	<0.001	<0.001	***

Sign. levels: *** : p < 0.001, ** : p < 0.01, * : p < 0.05

Table S11. Kruskal–Wallis (KW) test and pairwise Dunn's (D) test with Bonferroni correction for p-values on the difference of the MAD pairwise angle difference of the settlement relationship types a, b, and c for every time step. The Dunn's test was exclusively utilized for time steps in which the p-value of the KW test was less than 0.05.

Time step	Type 1	Type 2	n 1	n 2	median 1	median 2	KW p-value	adj. D p-value	*sign.*
1899	a	b	1566	744	7.78	7.35	0.053		
1899	a	c	1566	831	7.78	7.43	0.053		
1899	b	c	744	831	7.35	7.43	0.053		
1918	a	b	458	2149	7.80	7.74	0.002	1	
1918	a	c	458	885	7.80	6.88	0.002	0.012	*
1918	b	c	2149	885	7.74	6.88	0.002	0.001	**
1933	a	b	414	2233	8.25	8.01	<0.001	0.394	
1933	a	c	414	1098	8.25	7.08	<0.001	<0.001	***
1933	b	c	2233	1098	8.01	7.08	<0.001	<0.001	***
1959	a	b	179	1525	9.12	9.12	<0.001	0.789	
1959	a	c	179	2068	9.12	8.21	<0.001	0.184	
1959	b	c	1525	2068	9.12	8.21	<0.001	<0.001	***
1970	a	b	176	1496	8.34	8.99	<0.001	0.369	
1970	a	c	176	2390	8.34	8.35	<0.001	0.953	
1970	b	c	1496	2390	8.99	8.35	<0.001	<0.001	***
1978	a	b	147	1562	8.73	8.85	0.007	1	
1978	a	c	147	2572	8.73	8.22	0.007	0.2	
1978	b	c	1562	2572	8.85	8.22	0.007	0.005	**
2020	a	b	50	538	8.34	10.00	<0.001	0.002	**
2020	a	c	50	3441	8.34	8.18	<0.001	0.253	
2020	b	c	538	3441	10.00	8.18	<0.001	<0.001	***

Sign. levels: *** : p < 0.001, ** : p < 0.01, * : p < 0.05

Table S12. Kruskal–Wallis (KW) test and pairwise Dunn's (D) test with Bonferroni correction for p-values on the difference of the MAD pairwise compactness difference of the settlement relationship types a, b, and c for every time step. The Dunn's test was exclusively utilized for time steps in which the p-value of the KW test was less than 0.05.

Time step	Type 1	Type 2	n 1	n 2	median 1	median 2	KW p-value	adj. D p-value	*sign.*
1899	a	b	1566	744	0.046	0.038	<0.001	<0.001	***
1899	a	c	1566	831	0.046	0.042	<0.001	<0.001	***
1899	b	c	744	831	0.038	0.042	<0.001	0.008	**
1918	a	b	458	2149	0.053	0.045	<0.001	<0.001	***
1918	a	c	458	885	0.053	0.043	<0.001	<0.001	***
1918	b	c	2149	885	0.045	0.043	<0.001	0.058	
1933	a	b	414	2233	0.059	0.049	<0.001	<0.001	***
1933	a	c	414	1098	0.059	0.041	<0.001	<0.001	***
1933	b	c	2233	1098	0.049	0.041	<0.001	<0.001	***
1959	a	b	179	1525	0.067	0.067	<0.001	0.963	
1959	a	c	179	2068	0.067	0.055	<0.001	<0.001	***
1959	b	c	1525	2068	0.067	0.055	<0.001	<0.001	***
1970	a	b	176	1496	0.071	0.070	<0.001	0.522	
1970	a	c	176	2390	0.071	0.059	<0.001	<0.001	***
1970	b	c	1496	2390	0.070	0.059	<0.001	<0.001	***
1978	a	b	147	1562	0.072	0.071	<0.001	0.761	
1978	a	c	147	2572	0.072	0.063	<0.001	0.009	**
1978	b	c	1562	2572	0.071	0.063	<0.001	<0.001	***
2020	a	b	50	538	0.140	0.134	<0.001	1	
2020	a	c	50	3441	0.140	0.124	<0.001	0.244	
2020	b	c	538	3441	0.134	0.124	<0.001	<0.001	***

Sign. levels: *** : $p < 0.001$, ** : $p < 0.01$, * : $p < 0.05$

Table S13. Kruskal–Wallis (KW) test and pairwise Dunn's (D) test with Bonferroni correction for p-values on the difference of the MAD pairwise elongation difference of the settlement relationship types a, b, and c for every time step. The Dunn's test was exclusively utilized for time steps in which the p-value of the KW test was less than 0.05.

Time step	Type 1	Type 2	n 1	n 2	median 1	median 2	KW p-value	adj. D p-value	*sign.*
1899	a	b	1566	744	0.139	0.130	<0.001	<0.001	***
1899	a	c	1566	831	0.139	0.136	<0.001	0.013	*
1899	b	c	744	831	0.130	0.136	<0.001	0.002	**
1918	a	b	458	2149	0.142	0.137	<0.001	0.018	*
1918	a	c	458	885	0.142	0.134	<0.001	<0.001	***
1918	b	c	2149	885	0.137	0.134	<0.001	0.014	*
1933	a	b	414	2233	0.140	0.137	<0.001	0.052	
1933	a	c	414	1098	0.140	0.129	<0.001	<0.001	***
1933	b	c	2233	1098	0.137	0.129	<0.001	<0.001	***
1959	a	b	179	1525	0.153	0.150	<0.001	0.375	
1959	a	c	179	2068	0.153	0.145	<0.001	0.006	**
1959	b	c	1525	2068	0.150	0.145	<0.001	<0.001	***
1970	a	b	176	1496	0.156	0.150	<0.001	0.075	
1970	a	c	176	2390	0.156	0.146	<0.001	0.002	**
1970	b	c	1496	2390	0.150	0.146	<0.001	0.007	**
1978	a	b	147	1562	0.155	0.151	<0.001	0.201	
1978	a	c	147	2572	0.155	0.147	<0.001	0.005	**
1978	b	c	1562	2572	0.151	0.147	<0.001	<0.001	***
2020	a	b	50	538	0.174	0.168	0.922		
2020	a	c	50	3441	0.174	0.170	0.922		
2020	b	c	538	3441	0.168	0.170	0.922		

Sign. levels: *** : p < 0.001, ** : p < 0.01, * : p < 0.05

Table S14. Kruskal–Wallis (KW) test and pairwise Dunn's (D) test with Bonferroni correction for p-values on the difference of the MAD pairwise shape index difference of the settlement relationship types a, b, and c for every time step. The Dunn's test was exclusively utilized for time steps in which the p-value of the KW test was less than 0.05.

Time step	Type 1	Type 2	n 1	n 2	median 1	median 2	KW p-value	adj. D p-value	*sign.*
1899	a	b	1566	744	0.134	0.107	<0.001	<0.001	***
1899	a	c	1566	831	0.134	0.122	<0.001	<0.001	***
1899	b	c	744	831	0.107	0.122	<0.001	0.017	*
1918	a	b	458	2149	0.152	0.130	<0.001	<0.001	***
1918	a	c	458	885	0.152	0.121	<0.001	<0.001	***
1918	b	c	2149	885	0.130	0.121	<0.001	0.051	
1933	a	b	414	2233	0.174	0.142	<0.001	<0.001	***
1933	a	c	414	1098	0.174	0.114	<0.001	<0.001	***
1933	b	c	2233	1098	0.142	0.114	<0.001	<0.001	***
1959	a	b	179	1525	0.197	0.204	<0.001	1	
1959	a	c	179	2066	0.197	0.161	<0.001	<0.001	***
1959	b	c	1525	2066	0.204	0.161	<0.001	<0.001	***
1970	a	b	176	1496	0.218	0.210	<0.001	0.493	
1970	a	c	176	2386	0.218	0.175	<0.001	<0.001	***
1970	b	c	1496	2386	0.210	0.175	<0.001	<0.001	***
1978	a	b	147	1561	0.224	0.215	<0.001	0.789	
1978	a	c	147	2571	0.224	0.189	<0.001	0.011	*
1978	b	c	1561	2571	0.215	0.189	<0.001	<0.001	***
2020	a	b	50	538	0.534	0.476	0.004	0.102	
2020	a	c	50	3441	0.534	0.449	0.004	0.014	*
2020	b	c	538	3441	0.476	0.449	0.004	0.044	*

Sign. levels: *** : $p < 0.001$, ** : $p < 0.01$, * : $p < 0.05$

Analysis on the significant differences of the median of the median distributions of the pairwise differences per settlement relationship type

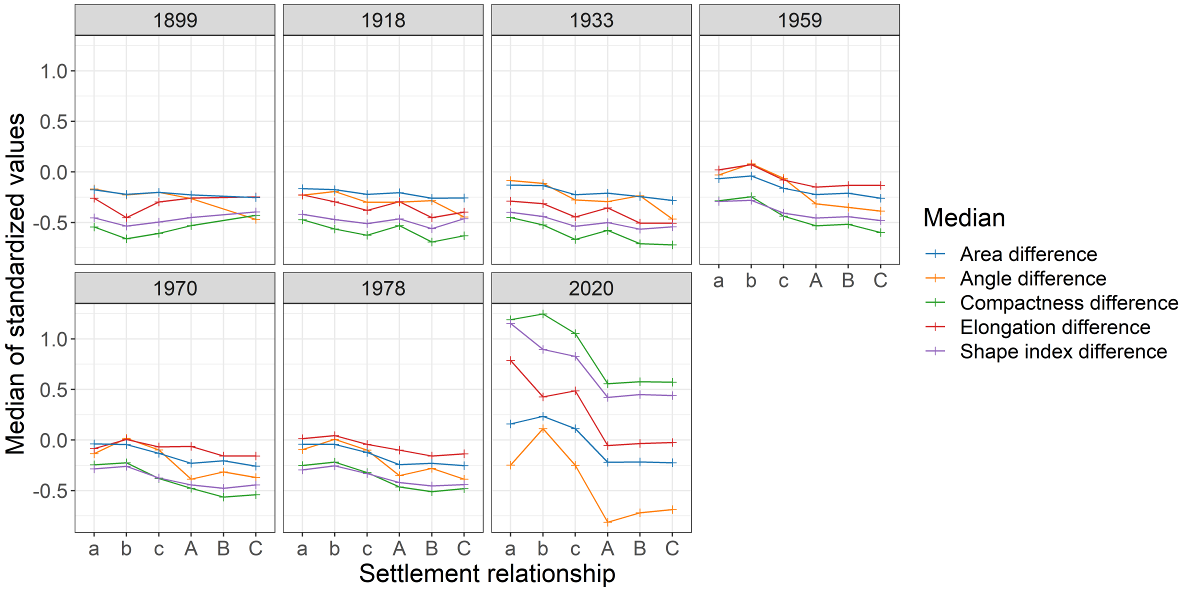

Figure S6. Median of the median distribution of urban morphometric differences per building pair for settlement relationships per time step: Lower median values indicate greater intra-settlement homogeneity. The values have been standardized in order to improve the clarity of the figure and facilitate comparison of trends.

Table S15. Kruskal–Wallis (KW) test and pairwise Dunn's (D) test with Bonferroni correction for p-values on the difference of the median pairwise area difference of the settlement relationship types a, b, and c for every time step. The Dunn's test was exclusively utilized for time steps in which the p-value of the KW test was less than 0.05.

Time step	Type 1	Type 2	n 1	n 2	median 1	median 2	KW p-value	adj. D p-value	*sign.*
1899	a	b	1566	744	100	89	<0.001	<0.001	***
1899	a	c	1566	831	100	94	<0.001	<0.001	***
1899	b	c	744	831	89	94	<0.001	0.045	*
1918	a	b	458	2149	103	100	<0.001	0.052	
1918	a	c	458	885	103	89	<0.001	<0.001	***
1918	b	c	2149	885	100	89	<0.001	<0.001	***
1933	a	b	414	2233	111	110	<0.001	0.572	
1933	a	c	414	1098	111	88	<0.001	<0.001	***
1933	b	c	2233	1098	110	88	<0.001	<0.001	***
1959	a	b	179	1525	126	133	<0.001	1	
1959	a	c	179	2068	126	104	<0.001	<0.001	***
1959	b	c	1525	2068	133	104	<0.001	<0.001	***
1970	a	b	176	1496	133	132	<0.001	1	
1970	a	c	176	2390	133	111	<0.001	<0.001	***
1970	b	c	1496	2390	132	111	<0.001	<0.001	***
1978	a	b	147	1562	132	132	<0.001	1	
1978	a	c	147	2572	132	113	<0.001	0.013	*
1978	b	c	1562	2572	132	113	<0.001	<0.001	***
2020	a	b	50	538	181	199	<0.001	0.665	
2020	a	c	50	3441	181	169	<0.001	0.02	*
2020	b	c	538	3441	199	169	<0.001	<0.001	***

Sign. levels: *** : p < 0.001, ** : p < 0.01, * : p < 0.05

Table S16. Kruskal–Wallis (KW) test and pairwise Dunn's (D) test with Bonferroni correction for p-values on the difference of the median pairwise angle difference of the settlement relationship types a, b, and c for every time step. The Dunn's test was exclusively utilized for time steps in which the p-value of the KW test was less than 0.05.

Time step	Type 1	Type 2	n 1	n 2	median 1	median 2	KW p-value	adj. D p-value	*sign.*
1899	a	b	1566	744	7.75	7.46	0.732		
1899	a	c	1566	831	7.75	7.59	0.732		
1899	b	c	744	831	7.46	7.59	0.732		
1918	a	b	458	2149	7.45	7.63	0.01	0.381	
1918	a	c	458	885	7.45	7.11	0.01	0.439	
1918	b	c	2149	885	7.63	7.11	0.01	0.004	**
1933	a	b	414	2233	8.16	8.02	<0.001	1	
1933	a	c	414	1098	8.16	7.22	<0.001	0.005	**
1933	b	c	2233	1098	8.02	7.22	<0.001	<0.001	***
1959	a	b	179	1525	8.43	8.96	<0.001	0.155	
1959	a	c	179	2068	8.43	8.26	<0.001	1	
1959	b	c	1525	2068	8.96	8.26	<0.001	<0.001	***
1970	a	b	176	1496	7.91	8.66	<0.001	0.061	
1970	a	c	176	2390	7.91	8.09	<0.001	0.85	
1970	b	c	1496	2390	8.66	8.09	<0.001	<0.001	***
1978	a	b	147	1562	8.11	8.61	0.048	0.816	
1978	a	c	147	2572	8.11	8.08	0.048	1	
1978	b	c	1562	2572	8.61	8.08	0.048	0.02	*
2020	a	b	50	538	7.36	9.12	<0.001	<0.001	***
2020	a	c	50	3441	7.36	7.35	<0.001	0.1	
2020	b	c	538	3441	9.12	7.35	<0.001	<0.001	***

Sign. levels: *** : $p < 0.001$, ** : $p < 0.01$, * : $p < 0.05$

Table S17. Kruskal–Wallis (KW) test and pairwise Dunn's (D) test with Bonferroni correction for p-values on the difference of the median pairwise compactness difference of the settlement relationship types a, b, and c for every time step. The Dunn's test was exclusively utilized for time steps in which the p-value of the KW test was less than 0.05.

Time step	Type 1	Type 2	n 1	n 2	median 1	median 2	KW p-value	adj. D p-value	*sign.*
1899	a	b	1566	744	0.045	0.039	<0.001	<0.001	***
1899	a	c	1566	831	0.045	0.042	<0.001	0.005	**
1899	b	c	744	831	0.039	0.042	<0.001	0.052	
1918	a	b	458	2149	0.049	0.044	<0.001	<0.001	***
1918	a	c	458	885	0.049	0.041	<0.001	<0.001	***
1918	b	c	2149	885	0.044	0.041	<0.001	0.054	
1933	a	b	414	2233	0.051	0.047	<0.001	0.017	*
1933	a	c	414	1098	0.051	0.039	<0.001	<0.001	***
1933	b	c	2233	1098	0.047	0.039	<0.001	<0.001	***
1959	a	b	179	1525	0.060	0.062	<0.001	0.554	
1959	a	c	179	2068	0.060	0.051	<0.001	0.008	**
1959	b	c	1525	2068	0.062	0.051	<0.001	<0.001	***
1970	a	b	176	1496	0.062	0.063	<0.001	1	
1970	a	c	176	2390	0.062	0.054	<0.001	0.02	*
1970	b	c	1496	2390	0.063	0.054	<0.001	<0.001	***
1978	a	b	147	1562	0.061	0.063	<0.001	1	
1978	a	c	147	2572	0.061	0.058	<0.001	0.294	
1978	b	c	1562	2572	0.063	0.058	<0.001	<0.001	***
2020	a	b	50	538	0.141	0.144	0.06		
2020	a	c	50	3441	0.141	0.133	0.06		
2020	b	c	538	3441	0.144	0.133	0.06		

Sign. levels: *** : p < 0.001, ** : p < 0.01, * : p < 0.05

Table S18. Kruskal–Wallis (KW) test and pairwise Dunn's (D) test with Bonferroni correction for p-values on the difference of the median pairwise elongation difference of the settlement relationship types a, b, and c for every time step. The Dunn's test was exclusively utilized for time steps in which the p-value of the KW test was less than 0.05.

Time step	Type 1	Type 2	n 1	n 2	median 1	median 2	KW p-value	adj. D p-value	*sign.*
1899	a	b	1566	744	0.162	0.152	<0.001	<0.001	***
1899	a	c	1566	831	0.162	0.160	<0.001	0.82	
1899	b	c	744	831	0.152	0.160	<0.001	<0.001	***
1918	a	b	458	2149	0.163	0.160	0.153		
1918	a	c	458	885	0.163	0.156	0.153		
1918	b	c	2149	885	0.160	0.156	0.153		
1933	a	b	414	2233	0.160	0.159	<0.001	1	
1933	a	c	414	1098	0.160	0.153	<0.001	<0.001	***
1933	b	c	2233	1098	0.159	0.153	<0.001	<0.001	***
1959	a	b	179	1525	0.175	0.178	<0.001	1	
1959	a	c	179	2068	0.175	0.171	<0.001	0.212	
1959	b	c	1525	2068	0.178	0.171	<0.001	<0.001	***
1970	a	b	176	1496	0.170	0.175	0.011	1	
1970	a	c	176	2390	0.170	0.171	0.011	0.28	
1970	b	c	1496	2390	0.175	0.171	0.011	0.006	**
1978	a	b	147	1562	0.175	0.176	0.07		
1978	a	c	147	2572	0.175	0.172	0.07		
1978	b	c	1562	2572	0.176	0.172	0.07		
2020	a	b	50	538	0.213	0.195	0.469		
2020	a	c	50	3441	0.213	0.198	0.469		
2020	b	c	538	3441	0.195	0.198	0.469		

Sign. levels: *** : p < 0.001, ** : p < 0.01, * : p < 0.05

Table S19. Kruskal–Wallis (KW) test and pairwise Dunn's (D) test with Bonferroni correction for p-values on the difference of the median pairwise shape index difference of the settlement relationship types a, b, and c for every time step. The Dunn's test was exclusively utilized for time steps in which the p-value of the KW test was less than 0.05.

Time step	Type 1	Type 2	n 1	n 2	median 1	median 2	KW p-value	adj. D p-value	*sign.*
1899	a	b	1566	744	0.128	0.107	<0.001	<0.001	***
1899	a	c	1566	831	0.128	0.118	<0.001	0.003	**
1899	b	c	744	831	0.107	0.118	<0.001	0.075	
1918	a	b	448	2136	0.138	0.124	<0.001	<0.001	***
1918	a	c	448	878	0.138	0.114	<0.001	<0.001	***
1918	b	c	2136	878	0.124	0.114	<0.001	0.043	*
1933	a	b	403	2207	0.143	0.132	<0.001	0.02	*
1933	a	c	403	1089	0.143	0.107	<0.001	<0.001	***
1933	b	c	2207	1089	0.132	0.107	<0.001	<0.001	***
1959	a	b	169	1421	0.171	0.174	<0.001	1	
1959	a	c	169	1958	0.171	0.140	<0.001	0.002	**
1959	b	c	1421	1958	0.174	0.140	<0.001	<0.001	***
1970	a	b	157	1419	0.172	0.179	<0.001	1	
1970	a	c	157	2279	0.172	0.149	<0.001	0.017	*
1970	b	c	1419	2279	0.179	0.149	<0.001	<0.001	***
1978	a	b	126	1473	0.170	0.180	<0.001	1	
1978	a	c	126	2431	0.170	0.160	<0.001	0.202	
1978	b	c	1473	2431	0.180	0.160	<0.001	<0.001	***
2020	a	b	50	538	0.549	0.481	0.057		
2020	a	c	50	3441	0.549	0.463	0.057		
2020	b	c	538	3441	0.481	0.463	0.057		

Sign. levels: *** : $p < 0.001$, ** : $p < 0.01$, * : $p < 0.05$

Analysis on the significant differences of the spatial distribution medians per settlement relationship type

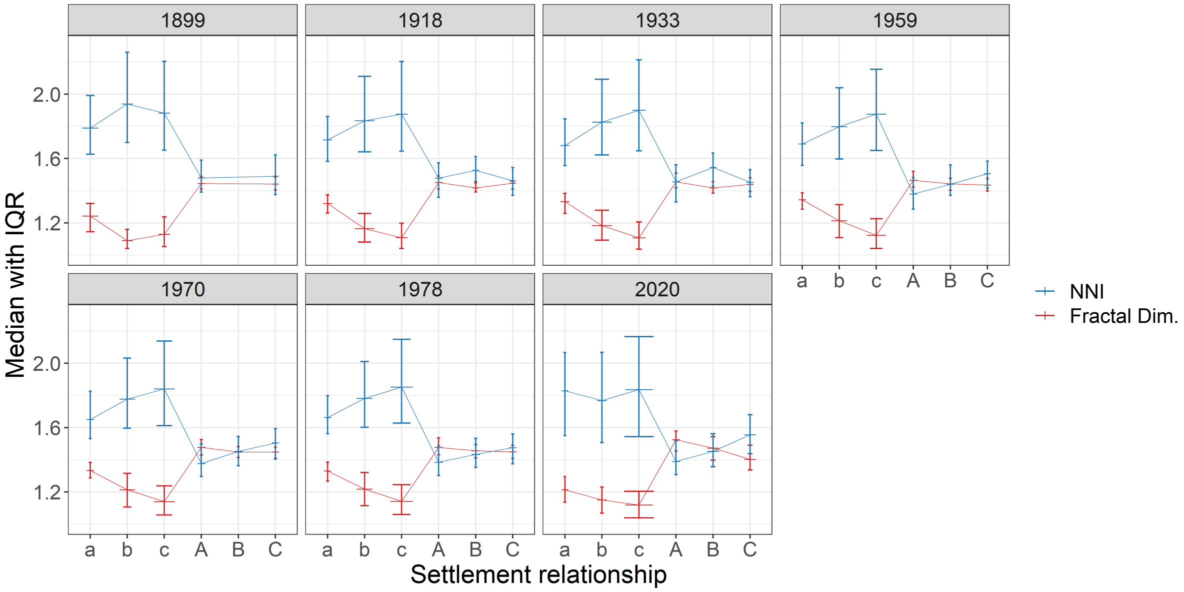

Figure S7. The distribution of the nearest neighbor index (NNI) and the fractal dimension for settlement relationships represented using the median and interquartile range (IQR) with varying width depending on the number of observations per relationship type. A lower fractal dimension implies less complexity and greater homogeneity in the dispersal of building footprints within a settlement. Conversely, a higher NNI suggests a greater regularity in the dispersal of building footprints.

Table S20. Kruskal–Wallis (KW) test and pairwise Dunn's (D) test with Bonferroni correction for p-values on the difference of the median nearest neighbor index of the settlement relationship types a, b, and c for every time step. The Dunn's test was exclusively utilized for time steps in which the p-value of the KW test was less than 0.05.

Time step	Type 1	Type 2	n 1	n 2	median 1	median 2	KW p-value	adj. D p-value	*sign.*
1899	a	b	1558	739	1.789	1.939	<0.001	<0.001	***
1899	a	c	1558	829	1.789	1.883	<0.001	<0.001	***
1899	b	c	739	829	1.939	1.883	<0.001	0.036	*
1918	a	b	457	2140	1.716	1.835	<0.001	<0.001	***
1918	a	c	457	881	1.716	1.876	<0.001	<0.001	***
1918	b	c	2140	881	1.835	1.876	<0.001	0.024	*
1933	a	b	414	2222	1.682	1.826	<0.001	<0.001	***
1933	a	c	414	1090	1.682	1.900	<0.001	<0.001	***
1933	b	c	2222	1090	1.826	1.900	<0.001	<0.001	***
1959	a	b	179	1517	1.691	1.798	<0.001	<0.001	***
1959	a	c	179	2059	1.691	1.876	<0.001	<0.001	***
1959	b	c	1517	2059	1.798	1.876	<0.001	<0.001	***
1970	a	b	176	1492	1.650	1.778	<0.001	<0.001	***
1970	a	c	176	2380	1.650	1.840	<0.001	<0.001	***
1970	b	c	1492	2380	1.778	1.840	<0.001	<0.001	***
1978	a	b	147	1556	1.663	1.782	<0.001	<0.001	***
1978	a	c	147	2560	1.663	1.852	<0.001	<0.001	***
1978	b	c	1556	2560	1.782	1.852	<0.001	<0.001	***
2020	a	b	50	537	1.829	1.768	0.007	0.866	
2020	a	c	50	3418	1.829	1.836	0.007	0.982	
2020	b	c	537	3418	1.768	1.836	0.007	0.002	**

Sign. levels: *** : p < 0.001, ** : p < 0.01, * : p < 0.05

Table S21. Kruskal–Wallis (KW) test and pairwise Dunn's (D) test with Bonferroni correction for p-values on the difference of the median fractal dimension of the settlement relationship types a, b, and c for every time step. The Dunn's test was exclusively utilized for time steps in which the p-value of the KW test was less than 0.05.

Time step	Type 1	Type 2	n 1	n 2	median 1	median 2	KW p-value	adj. D p-value	*sign.*
1899	a	b	1566	744	1.243	1.089	<0.001	<0.001	***
1899	a	c	1566	831	1.243	1.129	<0.001	<0.001	***
1899	b	c	744	831	1.089	1.129	<0.001	<0.001	***
1918	a	b	458	2149	1.321	1.164	<0.001	<0.001	***
1918	a	c	458	885	1.321	1.108	<0.001	<0.001	***
1918	b	c	2149	885	1.164	1.108	<0.001	<0.001	***
1933	a	b	414	2233	1.332	1.183	<0.001	<0.001	***
1933	a	c	414	1098	1.332	1.108	<0.001	<0.001	***
1933	b	c	2233	1098	1.183	1.108	<0.001	<0.001	***
1959	a	b	179	1525	1.343	1.213	<0.001	<0.001	***
1959	a	c	179	2068	1.343	1.123	<0.001	<0.001	***
1959	b	c	1525	2068	1.213	1.123	<0.001	<0.001	***
1970	a	b	176	1496	1.334	1.213	<0.001	<0.001	***
1970	a	c	176	2390	1.334	1.140	<0.001	<0.001	***
1970	b	c	1496	2390	1.213	1.140	<0.001	<0.001	***
1978	a	b	147	1562	1.331	1.218	<0.001	<0.001	***
1978	a	c	147	2572	1.331	1.142	<0.001	<0.001	***
1978	b	c	1562	2572	1.218	1.142	<0.001	<0.001	***
2020	a	b	50	538	1.214	1.151	<0.001	0.01	*
2020	a	c	50	3441	1.214	1.119	<0.001	<0.001	***
2020	b	c	538	3441	1.151	1.119	<0.001	<0.001	***

Sign. levels: *** : p < 0.001, ** : p < 0.01, * : p < 0.05

Number of settlements per time step

Table S22. Number of settlements per time step.

Time step	n
1899	3,346
1918	3,782
1933	4,116
1959	4,230
1970	4,685
1978	5,058
2020	6,145

Growing settlements

In Fig. S8 we can have a closer look on the distribution of buildings per settlement and time step. . Also can be seen is that there is an increase of the main summary statistics (mean, median, max) of the number of buildings per settlements over time.

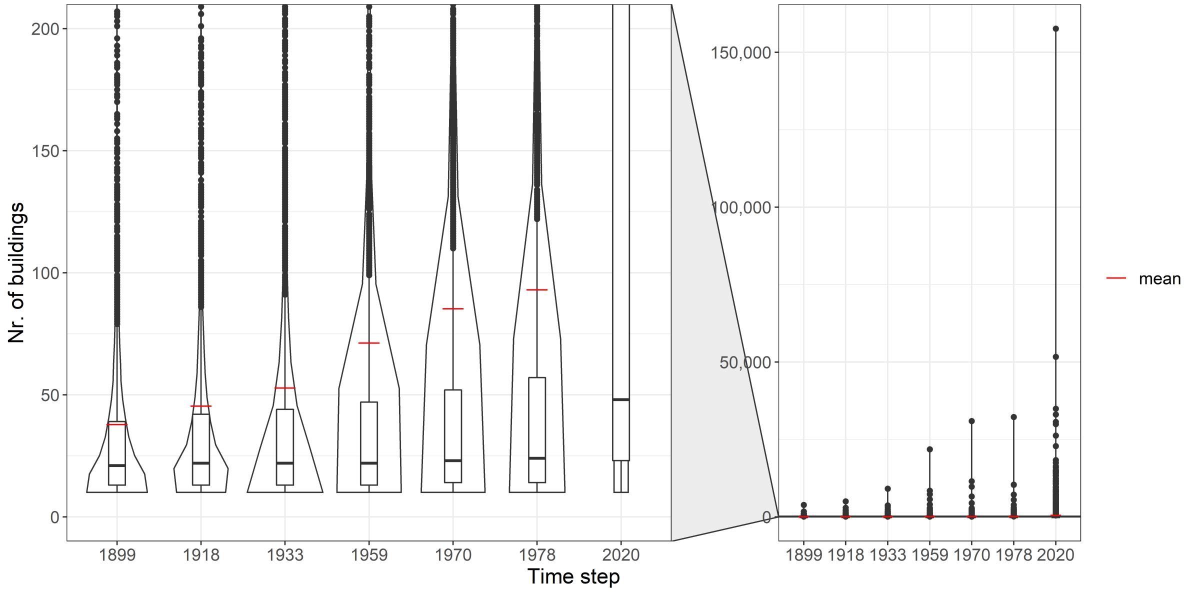

Figure S8. Violin plot and box plot of the number of buildings per settlement and time step. The settlements are increasing with regard to their number of houses over the years.

Relationship between settlement size and settlement relationship

Figure S9 shows the relationship between the settlement relationship and settlement size. Categories a and A have on average larger settlements, which is to be expected, as their definition is that they are the largest settlements, surrounded by smaller settlements.

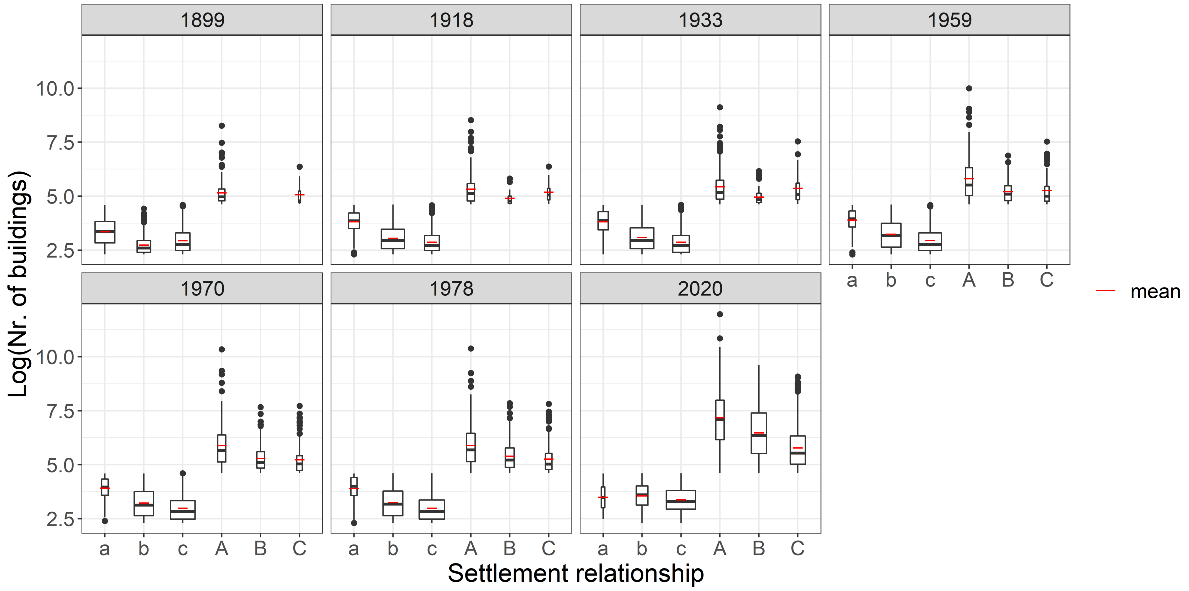

Figure S9. Distribution of the number of buildings per settlement relationship type and time step

Sensitivity analysis

In Tab. S65 to Tab. S92 we show the different significance and mean shifts due to the changes of the threshold of the settlement relationship category. For the various sensitivity analysis (SA) we shift the threshold that defines smaller and larger settlements (Nr. of buildings), as well as φ threshold that separates b from c (and B from C). We did not shift the upper threshold, the one separating a from b (and A from B) as a/A is by definition a settlement that is larger than its neighbor. A shift in that value away from 0 would lead to meaningless interpretations. We focused in the sensitivity analysis on the IQR distributions of the pairwise difference, as well as the median spatial distribution metrics. Table. S23 provides an overview of the different sensitivity analysis.

Table S23. Overview of the conducted sensitivity analysis. We focused in the sensitivity analysis on the IQR distributions of the pairwise difference, as well as the median spatial distribution metrics.

Scenario	Nr. of buildings	φ	Table
Base	100	-400	
SA1	100	-480	Tab. S65-S71
SA2	100	-320	Tab. S72-S78
SA3	120	-400	Tab. S79-S85
SA4	80	-400	Tab. S86-S92

SA1

Table S24. Kruskal–Wallis (KW) test and pairwise Dunn's (D) test with Bonferroni correction for p-values on the difference of the IQR pairwise area difference of the settlement relationship types a, b, and c for every time step. The Dunn's test was exclusively utilized for time steps in which the p-value of the KW test was less than 0.05.

Time step	Type 1	Type 2	n 1	n 2	median 1	median 2	KW p-value	adj. D p-value	*sign.*
1899	a	b	1566	767	127.250	108.250	<0.001	<0.001	***
1899	a	c	1566	808	127.250	123.875	<0.001	0.003	**
1899	b	c	767	808	108.250	123.875	<0.001	<0.001	***
1918	a	b	458	2254	139.750	128.500	<0.001	<0.001	***
1918	a	c	458	780	139.750	113.000	<0.001	<0.001	***
1918	b	c	2254	780	128.500	113.000	<0.001	<0.001	***
1933	a	b	414	2353	156.125	140.500	<0.001	<0.001	***
1933	a	c	414	978	156.125	114.375	<0.001	<0.001	***
1933	b	c	2353	978	140.500	114.375	<0.001	<0.001	***
1959	a	b	179	1685	180.500	177.500	<0.001	0.091	
1959	a	c	179	1908	180.500	141.000	<0.001	<0.001	***
1959	b	c	1685	1908	177.500	141.000	<0.001	<0.001	***
1970	a	b	176	1623	195.875	187.500	<0.001	0.209	
1970	a	c	176	2263	195.875	157.500	<0.001	<0.001	***
1970	b	c	1623	2263	187.500	157.500	<0.001	<0.001	***
1978	a	b	147	1691	212.000	199.000	<0.001	0.136	
1978	a	c	147	2443	212.000	171.000	<0.001	<0.001	***
1978	b	c	1691	2443	199.000	171.000	<0.001	<0.001	***
2020	a	b	50	613	383.344	322.415	<0.001	0.203	
2020	a	c	50	3366	383.344	273.852	<0.001	0.002	**
2020	b	c	613	3366	322.415	273.852	<0.001	<0.001	***

Sign. levels: *** : p < 0.001, ** : p < 0.01, * : p < 0.05

Table S25. Kruskal–Wallis (KW) test and pairwise Dunn's (D) test with Bonferroni correction for p-values on the difference of the IQR pairwise angle difference of the settlement relationship types a, b, and c for every time step. The Dunn's test was exclusively utilized for time steps in which the p-value of the KW test was less than 0.05.

Time step	Type 1	Type 2	n 1	n 2	median 1	median 2	KW p-value	adj. D p-value	*sign.*
1899	a	b	1566	767	13.242	11.960	0.004	0.005	**
1899	a	c	1566	808	13.242	12.584	0.004	0.02	*
1899	b	c	767	808	11.960	12.584	0.004	0.999	
1918	a	b	458	2254	13.494	13.068	<0.001	0.414	
1918	a	c	458	780	13.494	11.707	<0.001	0.002	**
1918	b	c	2254	780	13.068	11.707	<0.001	0.002	**
1933	a	b	414	2353	13.750	13.210	<0.001	0.29	
1933	a	c	414	978	13.750	12.001	<0.001	<0.001	***
1933	b	c	2353	978	13.210	12.001	<0.001	<0.001	***
1959	a	b	179	1685	14.950	14.945	<0.001	0.999	
1959	a	c	179	1908	14.950	13.883	<0.001	0.33	
1959	b	c	1685	1908	14.945	13.883	<0.001	<0.001	***
1970	a	b	176	1623	14.238	14.635	0.015	0.781	
1970	a	c	176	2263	14.238	14.055	0.015	0.872	
1970	b	c	1623	2263	14.635	14.055	0.015	0.006	**
1978	a	b	147	1691	15.460	14.410	0.012	0.279	
1978	a	c	147	2443	15.460	14.055	0.012	0.042	*
1978	b	c	1691	2443	14.410	14.055	0.012	0.032	*
2020	a	b	50	613	13.608	16.298	<0.001	0.009	**
2020	a	c	50	3366	13.608	14.232	<0.001	0.273	
2020	b	c	613	3366	16.298	14.232	<0.001	<0.001	***

Sign. levels: *** : p < 0.001, ** : p < 0.01, * : p < 0.05

Table S26. Kruskal–Wallis (KW) test and pairwise Dunn's (D) test with Bonferroni correction for p-values on the difference of the IQR pairwise compactness difference of the settlement relationship types a, b, and c for every time step. The Dunn's test was exclusively utilized for time steps in which the p-value of the KW test was less than 0.05.

Time step	Type 1	Type 2	n 1	n 2	median 1	median 2	KW p-value	adj. D p-value	*sign.*
1899	a	b	1566	767	0.077	0.062	<0.001	<0.001	***
1899	a	c	1566	808	0.077	0.073	<0.001	0.055	
1899	b	c	767	808	0.062	0.073	<0.001	<0.001	***
1918	a	b	458	2254	0.096	0.077	<0.001	<0.001	***
1918	a	c	458	780	0.096	0.074	<0.001	<0.001	***
1918	b	c	2254	780	0.077	0.074	<0.001	0.547	
1933	a	b	414	2353	0.105	0.086	<0.001	<0.001	***
1933	a	c	414	978	0.105	0.073	<0.001	<0.001	***
1933	b	c	2353	978	0.086	0.073	<0.001	<0.001	***
1959	a	b	179	1685	0.119	0.113	<0.001	0.153	
1959	a	c	179	1908	0.119	0.093	<0.001	<0.001	***
1959	b	c	1685	1908	0.113	0.093	<0.001	<0.001	***
1970	a	b	176	1623	0.117	0.118	<0.001	0.449	
1970	a	c	176	2263	0.117	0.104	<0.001	<0.001	***
1970	b	c	1623	2263	0.118	0.104	<0.001	<0.001	***
1978	a	b	147	1691	0.126	0.121	<0.001	0.439	
1978	a	c	147	2443	0.126	0.110	<0.001	0.007	**
1978	b	c	1691	2443	0.121	0.110	<0.001	<0.001	***
2020	a	b	50	613	0.201	0.190	<0.001	1	
2020	a	c	50	3366	0.201	0.178	<0.001	0.373	
2020	b	c	613	3366	0.190	0.178	<0.001	<0.001	***

Sign. levels: *** : $p < 0.001$, ** : $p < 0.01$, * : $p < 0.05$

Table S27. Kruskal–Wallis (KW) test and pairwise Dunn's (D) test with Bonferroni correction for p-values on the difference of the IQR pairwise elongation difference of the settlement relationship types a, b, and c for every time step. The Dunn's test was exclusively utilized for time steps in which the p-value of the KW test was less than 0.05.

Time step	Type 1	Type 2	n 1	n 2	median 1	median 2	KW p-value	adj. D p-value	*sign.*
1899	a	b	1566	767	0.194	0.181	<0.001	<0.001	***
1899	a	c	1566	808	0.194	0.188	<0.001	0.01	*
1899	b	c	767	808	0.181	0.188	<0.001	0.006	**
1918	a	b	458	2254	0.199	0.191	<0.001	0.002	**
1918	a	c	458	780	0.199	0.187	<0.001	<0.001	***
1918	b	c	2254	780	0.191	0.187	<0.001	0.099	
1933	a	b	414	2353	0.199	0.190	<0.001	0.001	**
1933	a	c	414	978	0.199	0.180	<0.001	<0.001	***
1933	b	c	2353	978	0.190	0.180	<0.001	<0.001	***
1959	a	b	179	1685	0.212	0.206	<0.001	0.122	
1959	a	c	179	1908	0.212	0.200	<0.001	0.002	**
1959	b	c	1685	1908	0.206	0.200	<0.001	0.001	**
1970	a	b	176	1623	0.214	0.207	<0.001	0.02	*
1970	a	c	176	2263	0.214	0.201	<0.001	<0.001	***
1970	b	c	1623	2263	0.207	0.201	<0.001	0.003	**
1978	a	b	147	1691	0.212	0.207	<0.001	0.152	
1978	a	c	147	2443	0.212	0.202	<0.001	0.004	**
1978	b	c	1691	2443	0.207	0.202	<0.001	<0.001	***
2020	a	b	50	613	0.249	0.237	0.916		
2020	a	c	50	3366	0.249	0.236	0.916		
2020	b	c	613	3366	0.237	0.236	0.916		

Sign. levels: *** : p < 0.001, ** : p < 0.01, * : p < 0.05

Table S28. Kruskal–Wallis (KW) test and pairwise Dunn's (D) test with Bonferroni correction for p-values on the difference of the IQR pairwise shape index difference of the settlement relationship types a, b, and c for every time step. The Dunn's test was exclusively utilized for time steps in which the p-value of the KW test was less than 0.05.

Time step	Type 1	Type 2	n 1	n 2	median 1	median 2	KW p-value	adj. D p-value	*sign.*
1899	a	b	1566	767	0.232	0.185	<0.001	<0.001	***
1899	a	c	1566	808	0.232	0.221	<0.001	0.062	
1899	b	c	767	808	0.185	0.221	<0.001	<0.001	***
1918	a	b	458	2254	0.307	0.238	<0.001	<0.001	***
1918	a	c	458	779	0.307	0.227	<0.001	<0.001	***
1918	b	c	2254	779	0.238	0.227	<0.001	0.542	
1933	a	b	414	2352	0.338	0.265	<0.001	<0.001	***
1933	a	c	414	977	0.338	0.222	<0.001	<0.001	***
1933	b	c	2352	977	0.265	0.222	<0.001	<0.001	***
1959	a	b	179	1665	0.378	0.364	<0.001	0.154	
1959	a	c	179	1877	0.378	0.290	<0.001	<0.001	***
1959	b	c	1665	1877	0.364	0.290	<0.001	<0.001	***
1970	a	b	176	1604	0.387	0.386	<0.001	0.307	
1970	a	c	176	2230	0.387	0.326	<0.001	<0.001	***
1970	b	c	1604	2230	0.386	0.326	<0.001	<0.001	***
1978	a	b	147	1671	0.409	0.391	<0.001	0.428	
1978	a	c	147	2392	0.409	0.346	<0.001	0.003	**
1978	b	c	1671	2392	0.391	0.346	<0.001	<0.001	***
2020	a	b	50	613	0.815	0.681	0.002	0.038	*
2020	a	c	50	3366	0.815	0.660	0.002	0.005	**
2020	b	c	613	3366	0.681	0.660	0.002	0.069	

Sign. levels: *** : $p < 0.001$, ** : $p < 0.01$, * : $p < 0.05$

Table S29. Kruskal–Wallis (KW) test and pairwise Dunn's (D) test with Bonferroni correction for p-values on the difference of the median nearest neighbor index of the settlement relationship types a, b, and c for every time step. The Dunn's test was exclusively utilized for time steps in which the p-value of the KW test was less than 0.05.

Time step	Type 1	Type 2	n 1	n 2	median 1	median 2	KW p-value	adj. D p-value	*sign.*
1899	a	b	1558	762	1.789	1.937	<0.001	<0.001	***
1899	a	c	1558	806	1.789	1.882	<0.001	<0.001	***
1899	b	c	762	806	1.937	1.882	<0.001	0.054	
1918	a	b	457	2245	1.716	1.838	<0.001	<0.001	***
1918	a	c	457	776	1.716	1.870	<0.001	<0.001	***
1918	b	c	2245	776	1.838	1.870	<0.001	0.167	
1933	a	b	414	2340	1.682	1.828	<0.001	<0.001	***
1933	a	c	414	972	1.682	1.900	<0.001	<0.001	***
1933	b	c	2340	972	1.828	1.900	<0.001	0.002	**
1959	a	b	179	1676	1.691	1.798	<0.001	<0.001	***
1959	a	c	179	1900	1.691	1.880	<0.001	<0.001	***
1959	b	c	1676	1900	1.798	1.880	<0.001	<0.001	***
1970	a	b	176	1617	1.650	1.784	<0.001	<0.001	***
1970	a	c	176	2255	1.650	1.838	<0.001	<0.001	***
1970	b	c	1617	2255	1.784	1.838	<0.001	<0.001	***
1978	a	b	147	1685	1.663	1.787	<0.001	<0.001	***
1978	a	c	147	2431	1.663	1.852	<0.001	<0.001	***
1978	b	c	1685	2431	1.787	1.852	<0.001	<0.001	***
2020	a	b	50	612	1.829	1.773	0.004	0.886	
2020	a	c	50	3343	1.829	1.837	0.004	0.962	
2020	b	c	612	3343	1.773	1.837	0.004	0.001	**

Sign. levels: *** : $p < 0.001$, ** : $p < 0.01$, * : $p < 0.05$

Table S30. Kruskal–Wallis (KW) test and pairwise Dunn's (D) test with Bonferroni correction for p-values on the difference of the median fractal dimension of the settlement relationship types a, b, and c for every time step. The Dunn's test was exclusively utilized for time steps in which the p-value of the KW test was less than 0.05.

Time step	Type 1	Type 2	n 1	n 2	median 1	median 2	KW p-value	adj. D p-value	*sign.*
1899	a	b	1566	767	1.243	1.089	<0.001	<0.001	***
1899	a	c	1566	808	1.243	1.130	<0.001	<0.001	***
1899	b	c	767	808	1.089	1.130	<0.001	<0.001	***
1918	a	b	458	2254	1.321	1.161	<0.001	<0.001	***
1918	a	c	458	780	1.321	1.109	<0.001	<0.001	***
1918	b	c	2254	780	1.161	1.109	<0.001	<0.001	***
1933	a	b	414	2353	1.332	1.177	<0.001	<0.001	***
1933	a	c	414	978	1.332	1.111	<0.001	<0.001	***
1933	b	c	2353	978	1.177	1.111	<0.001	<0.001	***
1959	a	b	179	1685	1.343	1.209	<0.001	<0.001	***
1959	a	c	179	1908	1.343	1.120	<0.001	<0.001	***
1959	b	c	1685	1908	1.209	1.120	<0.001	<0.001	***
1970	a	b	176	1623	1.334	1.212	<0.001	<0.001	***
1970	a	c	176	2263	1.334	1.139	<0.001	<0.001	***
1970	b	c	1623	2263	1.212	1.139	<0.001	<0.001	***
1978	a	b	147	1691	1.331	1.216	<0.001	<0.001	***
1978	a	c	147	2443	1.331	1.138	<0.001	<0.001	***
1978	b	c	1691	2443	1.216	1.138	<0.001	<0.001	***
2020	a	b	50	613	1.214	1.153	<0.001	0.012	*
2020	a	c	50	3366	1.214	1.118	<0.001	<0.001	***
2020	b	c	613	3366	1.153	1.118	<0.001	<0.001	***

Sign. levels: *** : p < 0.001, ** : p < 0.01, * : p < 0.05

SA2

Table S31. Kruskal–Wallis (KW) test and pairwise Dunn's (D) test with Bonferroni correction for p-values on the difference of the IQR pairwise area difference of the settlement relationship types a, b, and c for every time step. The Dunn's test was exclusively utilized for time steps in which the p-value of the KW test was less than 0.05.

Time step	Type 1	Type 2	n 1	n 2	median 1	median 2	KW p-value	adj. D p-value	*sign.*
1899	a	b	1566	722	127.250	108.625	<0.001	<0.001	***
1899	a	c	1566	853	127.250	123.000	<0.001	<0.001	***
1899	b	c	722	853	108.625	123.000	<0.001	<0.001	***
1918	a	b	458	2012	139.750	130.125	<0.001	<0.001	***
1918	a	c	458	1022	139.750	112.625	<0.001	<0.001	***
1918	b	c	2012	1022	130.125	112.625	<0.001	<0.001	***
1933	a	b	414	2052	156.125	142.625	<0.001	<0.001	***
1933	a	c	414	1279	156.125	115.500	<0.001	<0.001	***
1933	b	c	2052	1279	142.625	115.500	<0.001	<0.001	***
1959	a	b	179	1364	180.500	179.500	<0.001	0.189	
1959	a	c	179	2229	180.500	146.500	<0.001	<0.001	***
1959	b	c	1364	2229	179.500	146.500	<0.001	<0.001	***
1970	a	b	176	1340	195.875	188.000	<0.001	0.231	
1970	a	c	176	2546	195.875	161.125	<0.001	<0.001	***
1970	b	c	1340	2546	188.000	161.125	<0.001	<0.001	***
1978	a	b	147	1372	212.000	201.250	<0.001	0.218	
1978	a	c	147	2762	212.000	173.125	<0.001	<0.001	***
1978	b	c	1372	2762	201.250	173.125	<0.001	<0.001	***
2020	a	b	50	446	383.344	320.679	<0.001	0.22	
2020	a	c	50	3533	383.344	276.831	<0.001	0.003	**
2020	b	c	446	3533	320.679	276.831	<0.001	<0.001	***

Sign. levels: *** : p < 0.001, ** : p < 0.01, * : p < 0.05

Table S32. Kruskal–Wallis (KW) test and pairwise Dunn's (D) test with Bonferroni correction for p-values on the difference of the IQR pairwise angle difference of the settlement relationship types a, b, and c for every time step. The Dunn's test was exclusively utilized for time steps in which the p-value of the KW test was less than 0.05.

Time step	Type 1	Type 2	n 1	n 2	median 1	median 2	KW p-value	adj. D p-value	*sign.*
1899	a	b	1566	722	13.242	12.026	0.004	0.017	*
1899	a	c	1566	853	13.242	12.480	0.004	0.007	**
1899	b	c	722	853	12.026	12.480	0.004	1	
1918	a	b	458	2012	13.494	13.030	0.005	0.422	
1918	a	c	458	1022	13.494	12.106	0.005	0.007	**
1918	b	c	2012	1022	13.030	12.106	0.005	0.011	*
1933	a	b	414	2052	13.750	13.376	<0.001	0.452	
1933	a	c	414	1279	13.750	12.220	<0.001	<0.001	***
1933	b	c	2052	1279	13.376	12.220	<0.001	<0.001	***
1959	a	b	179	1364	14.950	14.949	0.002	0.934	
1959	a	c	179	2229	14.950	14.000	0.002	0.451	
1959	b	c	1364	2229	14.949	14.000	0.002	<0.001	***
1970	a	b	176	1340	14.238	14.720	0.021	0.709	
1970	a	c	176	2546	14.238	14.060	0.021	0.964	
1970	b	c	1340	2546	14.720	14.060	0.021	0.008	**
1978	a	b	147	1372	15.460	14.585	0.008	0.359	
1978	a	c	147	2762	15.460	14.014	0.008	0.045	*
1978	b	c	1372	2762	14.585	14.014	0.008	0.02	*
2020	a	b	50	446	13.608	16.751	<0.001	0.004	**
2020	a	c	50	3533	13.608	14.270	<0.001	0.257	
2020	b	c	446	3533	16.751	14.270	<0.001	<0.001	***

Sign. levels: *** : p < 0.001, ** : p < 0.01, * : p < 0.05

Table S33. Kruskal–Wallis (KW) test and pairwise Dunn's (D) test with Bonferroni correction for p-values on the difference of the IQR pairwise compactness difference of the settlement relationship types a, b, and c for every time step. The Dunn's test was exclusively utilized for time steps in which the p-value of the KW test was less than 0.05.

Time step	Type 1	Type 2	n 1	n 2	median 1	median 2	KW p-value	adj. D p-value	*sign.*
1899	a	b	1566	722	0.077	0.064	<0.001	<0.001	***
1899	a	c	1566	853	0.077	0.072	<0.001	0.016	*
1899	b	c	722	853	0.064	0.072	<0.001	0.006	**
1918	a	b	458	2012	0.096	0.079	<0.001	<0.001	***
1918	a	c	458	1022	0.096	0.071	<0.001	<0.001	***
1918	b	c	2012	1022	0.079	0.071	<0.001	0.003	**
1933	a	b	414	2052	0.105	0.087	<0.001	<0.001	***
1933	a	c	414	1279	0.105	0.072	<0.001	<0.001	***
1933	b	c	2052	1279	0.087	0.072	<0.001	<0.001	***
1959	a	b	179	1364	0.119	0.113	<0.001	0.227	
1959	a	c	179	2229	0.119	0.096	<0.001	<0.001	***
1959	b	c	1364	2229	0.113	0.096	<0.001	<0.001	***
1970	a	b	176	1340	0.117	0.117	<0.001	0.385	
1970	a	c	176	2546	0.117	0.106	<0.001	0.003	**
1970	b	c	1340	2546	0.117	0.106	<0.001	<0.001	***
1978	a	b	147	1372	0.126	0.120	<0.001	0.462	
1978	a	c	147	2762	0.126	0.112	<0.001	0.012	*
1978	b	c	1372	2762	0.120	0.112	<0.001	<0.001	***
2020	a	b	50	446	0.201	0.195	<0.001	1	
2020	a	c	50	3533	0.201	0.179	<0.001	0.384	
2020	b	c	446	3533	0.195	0.179	<0.001	<0.001	***

Sign. levels: *** : $p < 0.001$, ** : $p < 0.01$, * : $p < 0.05$

Table S34. Kruskal–Wallis (KW) test and pairwise Dunn's (D) test with Bonferroni correction for p-values on the difference of the IQR pairwise elongation difference of the settlement relationship types a, b, and c for every time step. The Dunn's test was exclusively utilized for time steps in which the p-value of the KW test was less than 0.05.

Time step	Type 1	Type 2	n 1	n 2	median 1	median 2	KW p-value	adj. D p-value	*sign.*
1899	a	b	1566	722	0.194	0.181	<0.001	<0.001	***
1899	a	c	1566	853	0.194	0.188	<0.001	0.004	**
1899	b	c	722	853	0.181	0.188	<0.001	0.011	*
1918	a	b	458	2012	0.199	0.191	<0.001	0.007	**
1918	a	c	458	1022	0.199	0.186	<0.001	<0.001	***
1918	b	c	2012	1022	0.191	0.186	<0.001	0.008	**
1933	a	b	414	2052	0.199	0.191	<0.001	0.006	**
1933	a	c	414	1279	0.199	0.180	<0.001	<0.001	***
1933	b	c	2052	1279	0.191	0.180	<0.001	<0.001	***
1959	a	b	179	1364	0.212	0.207	<0.001	0.154	
1959	a	c	179	2229	0.212	0.201	<0.001	0.003	**
1959	b	c	1364	2229	0.207	0.201	<0.001	0.003	**
1970	a	b	176	1340	0.214	0.208	<0.001	0.018	*
1970	a	c	176	2546	0.214	0.201	<0.001	<0.001	***
1970	b	c	1340	2546	0.208	0.201	<0.001	0.022	*
1978	a	b	147	1372	0.212	0.207	0.001	0.124	
1978	a	c	147	2762	0.212	0.203	0.001	0.007	**
1978	b	c	1372	2762	0.207	0.203	0.001	0.012	*
2020	a	b	50	446	0.249	0.240	0.763		
2020	a	c	50	3533	0.249	0.235	0.763		
2020	b	c	446	3533	0.240	0.235	0.763		

Sign. levels: *** : $p < 0.001$, ** : $p < 0.01$, * : $p < 0.05$

Table S35. Kruskal–Wallis (KW) test and pairwise Dunn's (D) test with Bonferroni correction for p-values on the difference of the IQR pairwise shape index difference of the settlement relationship types a, b, and c for every time step. The Dunn's test was exclusively utilized for time steps in which the p-value of the KW test was less than 0.05.

Time step	Type 1	Type 2	n 1	n 2	median 1	median 2	KW p-value	adj. D p-value	*sign.*
1899	a	b	1566	722	0.232	0.187	<0.001	<0.001	***
1899	a	c	1566	853	0.232	0.217	<0.001	0.02	*
1899	b	c	722	853	0.187	0.217	<0.001	0.003	**
1918	a	b	458	2012	0.307	0.245	<0.001	<0.001	***
1918	a	c	458	1021	0.307	0.218	<0.001	<0.001	***
1918	b	c	2012	1021	0.245	0.218	<0.001	0.003	**
1933	a	b	414	2052	0.338	0.270	<0.001	<0.001	***
1933	a	c	414	1277	0.338	0.218	<0.001	<0.001	***
1933	b	c	2052	1277	0.270	0.218	<0.001	<0.001	***
1959	a	b	179	1348	0.378	0.366	<0.001	0.235	
1959	a	c	179	2194	0.378	0.300	<0.001	<0.001	***
1959	b	c	1348	2194	0.366	0.300	<0.001	<0.001	***
1970	a	b	176	1325	0.387	0.384	<0.001	0.283	
1970	a	c	176	2509	0.387	0.332	<0.001	<0.001	***
1970	b	c	1325	2509	0.384	0.332	<0.001	<0.001	***
1978	a	b	147	1355	0.409	0.388	<0.001	0.443	
1978	a	c	147	2708	0.409	0.353	<0.001	0.006	**
1978	b	c	1355	2708	0.388	0.353	<0.001	<0.001	***
2020	a	b	50	446	0.815	0.687	<0.001	0.074	
2020	a	c	50	3533	0.815	0.660	<0.001	0.005	**
2020	b	c	446	3533	0.687	0.660	<0.001	0.021	*

Sign. levels: *** : p < 0.001, ** : p < 0.01, * : p < 0.05

Table S36. Kruskal–Wallis (KW) test and pairwise Dunn's (D) test with Bonferroni correction for p-values on the difference of the median nearest neighbor index of the settlement relationship types a, b, and c for every time step. The Dunn's test was exclusively utilized for time steps in which the p-value of the KW test was less than 0.05.

Time step	Type 1	Type 2	n 1	n 2	median 1	median 2	KW p-value	adj. D p-value	*sign.*
1899	a	b	1558	718	1.789	1.940	<0.001	<0.001	***
1899	a	c	1558	850	1.789	1.885	<0.001	<0.001	***
1899	b	c	718	850	1.940	1.885	<0.001	0.072	
1918	a	b	457	2004	1.716	1.837	<0.001	<0.001	***
1918	a	c	457	1017	1.716	1.870	<0.001	<0.001	***
1918	b	c	2004	1017	1.837	1.870	<0.001	0.042	*
1933	a	b	414	2043	1.682	1.822	<0.001	<0.001	***
1933	a	c	414	1269	1.682	1.904	<0.001	<0.001	***
1933	b	c	2043	1269	1.822	1.904	<0.001	<0.001	***
1959	a	b	179	1358	1.691	1.795	<0.001	<0.001	***
1959	a	c	179	2218	1.691	1.873	<0.001	<0.001	***
1959	b	c	1358	2218	1.795	1.873	<0.001	<0.001	***
1970	a	b	176	1336	1.650	1.773	<0.001	<0.001	***
1970	a	c	176	2536	1.650	1.840	<0.001	<0.001	***
1970	b	c	1336	2536	1.773	1.840	<0.001	<0.001	***
1978	a	b	147	1368	1.663	1.776	<0.001	<0.001	***
1978	a	c	147	2748	1.663	1.847	<0.001	<0.001	***
1978	b	c	1368	2748	1.776	1.847	<0.001	<0.001	***
2020	a	b	50	446	1.829	1.762	0.002	0.679	
2020	a	c	50	3509	1.829	1.835	0.002	0.982	
2020	b	c	446	3509	1.762	1.835	0.002	<0.001	***

Sign. levels: *** : p < 0.001, ** : p < 0.01, * : p < 0.05

Table S37. Kruskal–Wallis (KW) test and pairwise Dunn's (D) test with Bonferroni correction for p-values on the difference of the median fractal dimension of the settlement relationship types a, b, and c for every time step. The Dunn's test was exclusively utilized for time steps in which the p-value of the KW test was less than 0.05.

Time step	Type 1	Type 2	n 1	n 2	median 1	median 2	KW p-value	adj. D p-value	*sign.*
1899	a	b	1566	722	1.243	1.090	<0.001	<0.001	***
1899	a	c	1566	853	1.243	1.127	<0.001	<0.001	***
1899	b	c	722	853	1.090	1.127	<0.001	<0.001	***
1918	a	b	458	2012	1.321	1.169	<0.001	<0.001	***
1918	a	c	458	1022	1.321	1.108	<0.001	<0.001	***
1918	b	c	2012	1022	1.169	1.108	<0.001	<0.001	***
1933	a	b	414	2052	1.332	1.187	<0.001	<0.001	***
1933	a	c	414	1279	1.332	1.110	<0.001	<0.001	***
1933	b	c	2052	1279	1.187	1.110	<0.001	<0.001	***
1959	a	b	179	1364	1.343	1.222	<0.001	<0.001	***
1959	a	c	179	2229	1.343	1.125	<0.001	<0.001	***
1959	b	c	1364	2229	1.222	1.125	<0.001	<0.001	***
1970	a	b	176	1340	1.334	1.222	<0.001	<0.001	***
1970	a	c	176	2546	1.334	1.141	<0.001	<0.001	***
1970	b	c	1340	2546	1.222	1.141	<0.001	<0.001	***
1978	a	b	147	1372	1.331	1.223	<0.001	<0.001	***
1978	a	c	147	2762	1.331	1.144	<0.001	<0.001	***
1978	b	c	1372	2762	1.223	1.144	<0.001	<0.001	***
2020	a	b	50	446	1.214	1.150	<0.001	0.009	**
2020	a	c	50	3533	1.214	1.120	<0.001	<0.001	***
2020	b	c	446	3533	1.150	1.120	<0.001	<0.001	***

Sign. levels: *** : $p < 0.001$, ** : $p < 0.01$, * : $p < 0.05$

SA3

Table S38. Kruskal–Wallis (KW) test and pairwise Dunn's (D) test with Bonferroni correction for p-values on the difference of the IQR pairwise area difference of the settlement relationship types a, b, and c for every time step. The Dunn's test was exclusively utilized for time steps in which the p-value of the KW test was less than 0.05.

Time step	Type 1	Type 2	n 1	n 2	median 1	median 2	KW p-value	adj. D p-value	*sign.*
1899	a	b	1608	744	127.000	109.125	<0.001	<0.001	***
1899	a	c	1608	839	127.000	123.000	<0.001	<0.001	***
1899	b	c	744	839	109.125	123.000	<0.001	<0.001	***
1918	a	b	519	2172	140.000	128.500	<0.001	<0.001	***
1918	a	c	519	891	140.000	114.500	<0.001	<0.001	***
1918	b	c	2172	891	128.500	114.500	<0.001	<0.001	***
1933	a	b	457	2265	155.000	141.000	<0.001	<0.001	***
1933	a	c	457	1108	155.000	114.375	<0.001	<0.001	***
1933	b	c	2265	1108	141.000	114.375	<0.001	<0.001	***
1959	a	b	200	1569	175.000	179.000	<0.001	0.471	
1959	a	c	200	2091	175.000	142.250	<0.001	<0.001	***
1959	b	c	1569	2091	179.000	142.250	<0.001	<0.001	***
1970	a	b	195	1542	195.750	187.500	<0.001	0.158	
1970	a	c	195	2427	195.750	160.000	<0.001	<0.001	***
1970	b	c	1542	2427	187.500	160.000	<0.001	<0.001	***
1978	a	b	168	1614	204.500	200.000	<0.001	0.236	
1978	a	c	168	2616	204.500	171.000	<0.001	<0.001	***
1978	b	c	1614	2616	200.000	171.000	<0.001	<0.001	***
2020	a	b	53	587	386.338	313.165	<0.001	0.097	
2020	a	c	53	3550	386.338	271.809	<0.001	<0.001	***
2020	b	c	587	3550	313.165	271.809	<0.001	<0.001	***

Sign. levels: *** : p < 0.001, ** : p < 0.01, * : p < 0.05

Table S39. Kruskal–Wallis (KW) test and pairwise Dunn's (D) test with Bonferroni correction for p-values on the difference of the IQR pairwise angle difference of the settlement relationship types a, b, and c for every time step. The Dunn's test was exclusively utilized for time steps in which the p-value of the KW test was less than 0.05.

Time step	Type 1	Type 2	n 1	n 2	median 1	median 2	KW p-value	adj. D p-value	*sign.*
1899	a	b	1608	744	13.297	11.966	0.003	0.008	**
1899	a	c	1608	839	13.297	12.572	0.003	0.009	**
1899	b	c	744	839	11.966	12.572	0.003	1	
1918	a	b	519	2172	13.520	13.030	0.002	0.207	
1918	a	c	519	891	13.520	11.980	0.002	0.002	**
1918	b	c	2172	891	13.030	11.980	0.002	0.01	*
1933	a	b	457	2265	13.778	13.435	<0.001	0.325	
1933	a	c	457	1108	13.778	11.934	<0.001	<0.001	***
1933	b	c	2265	1108	13.435	11.934	<0.001	<0.001	***
1959	a	b	200	1569	14.982	14.950	0.001	1	
1959	a	c	200	2091	14.982	13.935	0.001	0.279	
1959	b	c	1569	2091	14.950	13.935	0.001	<0.001	***
1970	a	b	195	1542	14.240	14.591	0.021	0.712	
1970	a	c	195	2427	14.240	14.102	0.021	0.944	
1970	b	c	1542	2427	14.591	14.102	0.021	0.008	**
1978	a	b	168	1614	15.465	14.564	0.007	0.494	
1978	a	c	168	2616	15.465	14.041	0.007	0.06	
1978	b	c	1614	2616	14.564	14.041	0.007	0.012	*
2020	a	b	53	587	13.545	16.398	<0.001	0.007	**
2020	a	c	53	3550	13.545	14.230	<0.001	0.267	
2020	b	c	587	3550	16.398	14.230	<0.001	<0.001	***

Sign. levels: *** : p < 0.001, ** : p < 0.01, * : p < 0.05

Table S40. Kruskal–Wallis (KW) test and pairwise Dunn's (D) test with Bonferroni correction for p-values on the difference of the IQR pairwise compactness difference of the settlement relationship types a, b, and c for every time step. The Dunn's test was exclusively utilized for time steps in which the p-value of the KW test was less than 0.05.

Time step	Type 1	Type 2	n 1	n 2	median 1	median 2	KW p-value	adj. D p-value	*sign.*
1899	a	b	1608	744	0.077	0.063	<0.001	<0.001	***
1899	a	c	1608	839	0.077	0.072	<0.001	0.011	*
1899	b	c	744	839	0.063	0.072	<0.001	0.004	**
1918	a	b	519	2172	0.096	0.078	<0.001	<0.001	***
1918	a	c	519	891	0.096	0.073	<0.001	<0.001	***
1918	b	c	2172	891	0.078	0.073	<0.001	0.25	
1933	a	b	457	2265	0.103	0.086	<0.001	<0.001	***
1933	a	c	457	1108	0.103	0.072	<0.001	<0.001	***
1933	b	c	2265	1108	0.086	0.072	<0.001	<0.001	***
1959	a	b	200	1569	0.116	0.113	<0.001	0.29	
1959	a	c	200	2091	0.116	0.094	<0.001	<0.001	***
1959	b	c	1569	2091	0.113	0.094	<0.001	<0.001	***
1970	a	b	195	1542	0.123	0.118	<0.001	0.237	
1970	a	c	195	2427	0.123	0.105	<0.001	<0.001	***
1970	b	c	1542	2427	0.118	0.105	<0.001	<0.001	***
1978	a	b	168	1614	0.123	0.121	<0.001	0.548	
1978	a	c	168	2616	0.123	0.111	<0.001	0.008	**
1978	b	c	1614	2616	0.121	0.111	<0.001	<0.001	***
2020	a	b	53	587	0.205	0.189	<0.001	1	
2020	a	c	53	3550	0.205	0.177	<0.001	0.264	
2020	b	c	587	3550	0.189	0.177	<0.001	<0.001	***

Sign. levels: *** : $p < 0.001$, ** : $p < 0.01$, * : $p < 0.05$

Table S41. Kruskal–Wallis (KW) test and pairwise Dunn's (D) test with Bonferroni correction for p-values on the difference of the IQR pairwise elongation difference of the settlement relationship types a, b, and c for every time step. The Dunn's test was exclusively utilized for time steps in which the p-value of the KW test was less than 0.05.

Time step	Type 1	Type 2	n 1	n 2	median 1	median 2	KW p-value	adj. D p-value	*sign.*
1899	a	b	1608	744	0.194	0.181	<0.001	<0.001	***
1899	a	c	1608	839	0.194	0.188	<0.001	0.005	**
1899	b	c	744	839	0.181	0.188	<0.001	0.006	**
1918	a	b	519	2172	0.200	0.191	<0.001	<0.001	***
1918	a	c	519	891	0.200	0.187	<0.001	<0.001	***
1918	b	c	2172	891	0.191	0.187	<0.001	0.047	*
1933	a	b	457	2265	0.200	0.190	<0.001	0.001	**
1933	a	c	457	1108	0.200	0.179	<0.001	<0.001	***
1933	b	c	2265	1108	0.190	0.179	<0.001	<0.001	***
1959	a	b	200	1569	0.212	0.207	<0.001	0.118	
1959	a	c	200	2091	0.212	0.201	<0.001	0.001	**
1959	b	c	1569	2091	0.207	0.201	<0.001	0.001	**
1970	a	b	195	1542	0.216	0.208	<0.001	0.008	**
1970	a	c	195	2427	0.216	0.201	<0.001	<0.001	***
1970	b	c	1542	2427	0.208	0.201	<0.001	0.002	**
1978	a	b	168	1614	0.212	0.207	<0.001	0.179	
1978	a	c	168	2616	0.212	0.203	<0.001	0.004	**
1978	b	c	1614	2616	0.207	0.203	<0.001	<0.001	***
2020	a	b	53	587	0.254	0.235	0.815		
2020	a	c	53	3550	0.254	0.235	0.815		
2020	b	c	587	3550	0.235	0.235	0.815		

Sign. levels: *** : $p < 0.001$, ** : $p < 0.01$, * : $p < 0.05$

Table S42. Kruskal–Wallis (KW) test and pairwise Dunn's (D) test with Bonferroni correction for p-values on the difference of the IQR pairwise shape index difference of the settlement relationship types a, b, and c for every time step. The Dunn's test was exclusively utilized for time steps in which the p-value of the KW test was less than 0.05.

Time step	Type 1	Type 2	n 1	n 2	median 1	median 2	KW p-value	adj. D p-value	*sign.*
1899	a	b	1608	744	0.234	0.186	<0.001	<0.001	***
1899	a	c	1608	839	0.234	0.218	<0.001	0.013	*
1899	b	c	744	839	0.186	0.218	<0.001	0.003	**
1918	a	b	519	2172	0.304	0.241	<0.001	<0.001	***
1918	a	c	519	890	0.304	0.226	<0.001	<0.001	***
1918	b	c	2172	890	0.241	0.226	<0.001	0.256	
1933	a	b	457	2264	0.328	0.268	<0.001	<0.001	***
1933	a	c	457	1107	0.328	0.218	<0.001	<0.001	***
1933	b	c	2264	1107	0.268	0.218	<0.001	<0.001	***
1959	a	b	200	1551	0.368	0.366	<0.001	0.321	
1959	a	c	200	2058	0.368	0.293	<0.001	<0.001	***
1959	b	c	1551	2058	0.366	0.293	<0.001	<0.001	***
1970	a	b	195	1523	0.400	0.388	<0.001	0.129	
1970	a	c	195	2394	0.400	0.330	<0.001	<0.001	***
1970	b	c	1523	2394	0.388	0.330	<0.001	<0.001	***
1978	a	b	168	1595	0.408	0.391	<0.001	0.554	
1978	a	c	168	2564	0.408	0.349	<0.001	0.004	**
1978	b	c	1595	2564	0.391	0.349	<0.001	<0.001	***
2020	a	b	53	587	0.819	0.681	<0.001	0.029	*
2020	a	c	53	3550	0.819	0.657	<0.001	0.002	**
2020	b	c	587	3550	0.681	0.657	<0.001	0.029	*

Sign. levels: *** : p < 0.001, ** : p < 0.01, * : p < 0.05

Table S43. Kruskal–Wallis (KW) test and pairwise Dunn's (D) test with Bonferroni correction for p-values on the difference of the median nearest neighbor index of the settlement relationship types a, b, and c for every time step. The Dunn's test was exclusively utilized for time steps in which the p-value of the KW test was less than 0.05.

Time step	Type 1	Type 2	n 1	n 2	median 1	median 2	KW p-value	adj. D p-value	*sign.*
1899	a	b	1600	739	1.778	1.939	<0.001	<0.001	***
1899	a	c	1600	837	1.778	1.879	<0.001	<0.001	***
1899	b	c	739	837	1.939	1.879	<0.001	0.02	*
1918	a	b	518	2163	1.689	1.833	<0.001	<0.001	***
1918	a	c	518	887	1.689	1.874	<0.001	<0.001	***
1918	b	c	2163	887	1.833	1.874	<0.001	0.022	*
1933	a	b	457	2254	1.669	1.822	<0.001	<0.001	***
1933	a	c	457	1100	1.669	1.894	<0.001	<0.001	***
1933	b	c	2254	1100	1.822	1.894	<0.001	<0.001	***
1959	a	b	200	1561	1.674	1.788	<0.001	<0.001	***
1959	a	c	200	2082	1.674	1.872	<0.001	<0.001	***
1959	b	c	1561	2082	1.788	1.872	<0.001	<0.001	***
1970	a	b	195	1538	1.641	1.767	<0.001	<0.001	***
1970	a	c	195	2417	1.641	1.835	<0.001	<0.001	***
1970	b	c	1538	2417	1.767	1.835	<0.001	<0.001	***
1978	a	b	168	1608	1.651	1.770	<0.001	<0.001	***
1978	a	c	168	2604	1.651	1.843	<0.001	<0.001	***
1978	b	c	1608	2604	1.770	1.843	<0.001	<0.001	***
2020	a	b	53	586	1.827	1.754	<0.001	0.746	
2020	a	c	53	3527	1.827	1.824	<0.001	0.936	
2020	b	c	586	3527	1.754	1.824	<0.001	<0.001	***

Sign. levels: *** : p < 0.001, ** : p < 0.01, * : p < 0.05

Table S44. Kruskal–Wallis (KW) test and pairwise Dunn's (D) test with Bonferroni correction for p-values on the difference of the median fractal dimension of the settlement relationship types a, b, and c for every time step. The Dunn's test was exclusively utilized for time steps in which the p-value of the KW test was less than 0.05.

Time step	Type 1	Type 2	n 1	n 2	median 1	median 2	KW p-value	adj. D p-value	*sign.*
1899	a	b	1608	744	1.246	1.089	<0.001	<0.001	***
1899	a	c	1608	839	1.246	1.130	<0.001	<0.001	***
1899	b	c	744	839	1.089	1.130	<0.001	<0.001	***
1918	a	b	519	2172	1.335	1.166	<0.001	<0.001	***
1918	a	c	519	891	1.335	1.109	<0.001	<0.001	***
1918	b	c	2172	891	1.166	1.109	<0.001	<0.001	***
1933	a	b	457	2265	1.342	1.184	<0.001	<0.001	***
1933	a	c	457	1108	1.342	1.109	<0.001	<0.001	***
1933	b	c	2265	1108	1.184	1.109	<0.001	<0.001	***
1959	a	b	200	1569	1.348	1.221	<0.001	<0.001	***
1959	a	c	200	2091	1.348	1.125	<0.001	<0.001	***
1959	b	c	1569	2091	1.221	1.125	<0.001	<0.001	***
1970	a	b	195	1542	1.347	1.221	<0.001	<0.001	***
1970	a	c	195	2427	1.347	1.144	<0.001	<0.001	***
1970	b	c	1542	2427	1.221	1.144	<0.001	<0.001	***
1978	a	b	168	1614	1.341	1.226	<0.001	<0.001	***
1978	a	c	168	2616	1.341	1.145	<0.001	<0.001	***
1978	b	c	1614	2616	1.226	1.145	<0.001	<0.001	***
2020	a	b	53	587	1.231	1.163	<0.001	0.018	*
2020	a	c	53	3550	1.231	1.123	<0.001	<0.001	***
2020	b	c	587	3550	1.163	1.123	<0.001	<0.001	***

Sign. levels: *** : $p < 0.001$, ** : $p < 0.01$, * : $p < 0.05$

SA4

Table S45. Kruskal–Wallis (KW) test and pairwise Dunn's (D) test with Bonferroni correction for p-values on the difference of the IQR pairwise area difference of the settlement relationship types a, b, and c for every time step. The Dunn's test was exclusively utilized for time steps in which the p-value of the KW test was less than 0.05.

Time step	Type 1	Type 2	n 1	n 2	median 1	median 2	KW p-value	adj. D p-value	*sign.*
1899	a	b	1483	743	128.000	109.000	<0.001	<0.001	***
1899	a	c	1483	821	128.000	123.000	<0.001	<0.001	***
1899	b	c	743	821	109.000	123.000	<0.001	<0.001	***
1918	a	b	395	2123	140.500	129.000	<0.001	<0.001	***
1918	a	c	395	875	140.500	114.500	<0.001	<0.001	***
1918	b	c	2123	875	129.000	114.500	<0.001	<0.001	***
1933	a	b	351	2195	157.000	142.000	<0.001	<0.001	***
1933	a	c	351	1082	157.000	113.875	<0.001	<0.001	***
1933	b	c	2195	1082	142.000	113.875	<0.001	<0.001	***
1959	a	b	150	1458	184.250	179.500	<0.001	0.103	
1959	a	c	150	2044	184.250	142.000	<0.001	<0.001	***
1959	b	c	1458	2044	179.500	142.000	<0.001	<0.001	***
1970	a	b	138	1424	200.500	189.000	<0.001	0.129	
1970	a	c	138	2341	200.500	159.500	<0.001	<0.001	***
1970	b	c	1424	2341	189.000	159.500	<0.001	<0.001	***
1978	a	b	106	1479	223.625	201.750	<0.001	0.042	*
1978	a	c	106	2521	223.625	172.000	<0.001	<0.001	***
1978	b	c	1479	2521	201.750	172.000	<0.001	<0.001	***
2020	a	b	47	494	386.338	328.590	<0.001	0.243	
2020	a	c	47	3246	386.338	280.813	<0.001	0.005	**
2020	b	c	494	3246	328.590	280.813	<0.001	<0.001	***

Sign. levels: *** : p < 0.001, ** : p < 0.01, * : p < 0.05

Table S46. Kruskal–Wallis (KW) test and pairwise Dunn's (D) test with Bonferroni correction for p-values on the difference of the IQR pairwise angle difference of the settlement relationship types a, b, and c for every time step. The Dunn's test was exclusively utilized for time steps in which the p-value of the KW test was less than 0.05.

Time step	Type 1	Type 2	n 1	n 2	median 1	median 2	KW p-value	adj. D p-value	*sign.*
1899	a	b	1483	743	13.258	11.972	0.004	0.011	*
1899	a	c	1483	821	13.258	12.530	0.004	0.009	**
1899	b	c	743	821	11.972	12.530	0.004	1	
1918	a	b	395	2123	13.370	13.020	0.008	0.545	
1918	a	c	395	875	13.370	11.905	0.008	0.014	*
1918	b	c	2123	875	13.020	11.905	0.008	0.01	*
1933	a	b	351	2195	13.807	13.405	<0.001	0.542	
1933	a	c	351	1082	13.807	11.914	<0.001	<0.001	***
1933	b	c	2195	1082	13.405	11.914	<0.001	<0.001	***
1959	a	b	150	1458	15.062	14.943	0.002	1	
1959	a	c	150	2044	15.062	13.953	0.002	0.446	
1959	b	c	1458	2044	14.943	13.953	0.002	<0.001	***
1970	a	b	138	1424	14.020	14.636	0.033	1	
1970	a	c	138	2341	14.020	14.060	0.033	0.847	
1970	b	c	1424	2341	14.636	14.060	0.033	0.014	*
1978	a	b	106	1479	15.422	14.498	0.015	0.499	
1978	a	c	106	2521	15.422	13.972	0.015	0.107	
1978	b	c	1479	2521	14.498	13.972	0.015	0.02	*
2020	a	b	47	494	13.030	16.637	<0.001	0.002	**
2020	a	c	47	3246	13.030	14.279	<0.001	0.154	
2020	b	c	494	3246	16.637	14.279	<0.001	<0.001	***

Sign. levels: *** : p < 0.001, ** : p < 0.01, * : p < 0.05

Table S47. Kruskal–Wallis (KW) test and pairwise Dunn's (D) test with Bonferroni correction for p-values on the difference of the IQR pairwise compactness difference of the settlement relationship types a, b, and c for every time step. The Dunn's test was exclusively utilized for time steps in which the p-value of the KW test was less than 0.05.

Time step	Type 1	Type 2	n 1	n 2	median 1	median 2	KW p-value	adj. D p-value	*sign.*
1899	a	b	1483	743	0.076	0.063	<0.001	<0.001	***
1899	a	c	1483	821	0.076	0.072	<0.001	0.018	*
1899	b	c	743	821	0.063	0.072	<0.001	0.009	**
1918	a	b	395	2123	0.097	0.078	<0.001	<0.001	***
1918	a	c	395	875	0.097	0.073	<0.001	<0.001	***
1918	b	c	2123	875	0.078	0.073	<0.001	0.201	
1933	a	b	351	2195	0.102	0.087	<0.001	<0.001	***
1933	a	c	351	1082	0.102	0.071	<0.001	<0.001	***
1933	b	c	2195	1082	0.087	0.071	<0.001	<0.001	***
1959	a	b	150	1458	0.120	0.112	<0.001	0.238	
1959	a	c	150	2044	0.120	0.094	<0.001	<0.001	***
1959	b	c	1458	2044	0.112	0.094	<0.001	<0.001	***
1970	a	b	138	1424	0.123	0.118	<0.001	0.36	
1970	a	c	138	2341	0.123	0.105	<0.001	0.002	**
1970	b	c	1424	2341	0.118	0.105	<0.001	<0.001	***
1978	a	b	106	1479	0.137	0.120	<0.001	0.1	
1978	a	c	106	2521	0.137	0.111	<0.001	0.001	**
1978	b	c	1479	2521	0.120	0.111	<0.001	<0.001	***
2020	a	b	47	494	0.197	0.193	<0.001	1	
2020	a	c	47	3246	0.197	0.180	<0.001	0.559	
2020	b	c	494	3246	0.193	0.180	<0.001	<0.001	***

Sign. levels: *** : p < 0.001, ** : p < 0.01, * : p < 0.05

Table S48. Kruskal–Wallis (KW) test and pairwise Dunn's (D) test with Bonferroni correction for p-values on the difference of the IQR pairwise elongation difference of the settlement relationship types a, b, and c for every time step. The Dunn's test was exclusively utilized for time steps in which the p-value of the KW test was less than 0.05.

Time step	Type 1	Type 2	n 1	n 2	median 1	median 2	KW p-value	adj. D p-value	*sign.*
1899	a	b	1483	743	0.193	0.181	<0.001	<0.001	***
1899	a	c	1483	821	0.193	0.188	<0.001	0.011	*
1899	b	c	743	821	0.181	0.188	<0.001	0.011	*
1918	a	b	395	2123	0.200	0.191	<0.001	0.007	**
1918	a	c	395	875	0.200	0.187	<0.001	<0.001	***
1918	b	c	2123	875	0.191	0.187	<0.001	0.034	*
1933	a	b	351	2195	0.199	0.190	<0.001	0.016	*
1933	a	c	351	1082	0.199	0.179	<0.001	<0.001	***
1933	b	c	2195	1082	0.190	0.179	<0.001	<0.001	***
1959	a	b	150	1458	0.210	0.206	<0.001	0.195	
1959	a	c	150	2044	0.210	0.201	<0.001	0.006	**
1959	b	c	1458	2044	0.206	0.201	<0.001	0.002	**
1970	a	b	138	1424	0.217	0.208	<0.001	0.053	
1970	a	c	138	2341	0.217	0.201	<0.001	0.002	**
1970	b	c	1424	2341	0.208	0.201	<0.001	0.006	**
1978	a	b	106	1479	0.210	0.207	<0.001	0.288	
1978	a	c	106	2521	0.210	0.202	<0.001	0.02	*
1978	b	c	1479	2521	0.207	0.202	<0.001	<0.001	***
2020	a	b	47	494	0.254	0.238	0.932		
2020	a	c	47	3246	0.254	0.236	0.932		
2020	b	c	494	3246	0.238	0.236	0.932		

Sign. levels: *** : p < 0.001, ** : p < 0.01, * : p < 0.05

Table S49. Kruskal–Wallis (KW) test and pairwise Dunn's (D) test with Bonferroni correction for p-values on the difference of the IQR pairwise shape index difference of the settlement relationship types a, b, and c for every time step. The Dunn's test was exclusively utilized for time steps in which the p-value of the KW test was less than 0.05.

Time step	Type 1	Type 2	n 1	n 2	median 1	median 2	KW p-value	adj. D p-value	*sign.*
1899	a	b	1483	743	0.231	0.186	<0.001	<0.001	***
1899	a	c	1483	821	0.231	0.217	<0.001	0.02	*
1899	b	c	743	821	0.186	0.217	<0.001	0.006	**
1918	a	b	395	2123	0.314	0.241	<0.001	<0.001	***
1918	a	c	395	874	0.314	0.225	<0.001	<0.001	***
1918	b	c	2123	874	0.241	0.225	<0.001	0.205	
1933	a	b	351	2194	0.326	0.269	<0.001	<0.001	***
1933	a	c	351	1081	0.326	0.216	<0.001	<0.001	***
1933	b	c	2194	1081	0.269	0.216	<0.001	<0.001	***
1959	a	b	150	1440	0.371	0.363	<0.001	0.235	
1959	a	c	150	2011	0.371	0.293	<0.001	<0.001	***
1959	b	c	1440	2011	0.363	0.293	<0.001	<0.001	***
1970	a	b	138	1405	0.401	0.388	<0.001	0.187	
1970	a	c	138	2308	0.401	0.328	<0.001	<0.001	***
1970	b	c	1405	2308	0.388	0.328	<0.001	<0.001	***
1978	a	b	106	1460	0.445	0.388	<0.001	0.077	
1978	a	c	106	2469	0.445	0.348	<0.001	<0.001	***
1978	b	c	1460	2469	0.388	0.348	<0.001	<0.001	***
2020	a	b	47	494	0.839	0.685	0.002	0.051	
2020	a	c	47	3246	0.839	0.663	0.002	0.005	**
2020	b	c	494	3246	0.685	0.663	0.002	0.048	*

Sign. levels: *** : p < 0.001, ** : p < 0.01, * : p < 0.05

Table S50. Kruskal–Wallis (KW) test and pairwise Dunn's (D) test with Bonferroni correction for p-values on the difference of the median nearest neighbor index of the settlement relationship types a, b, and c for every time step. The Dunn's test was exclusively utilized for time steps in which the p-value of the KW test was less than 0.05.

Time step	Type 1	Type 2	n 1	n 2	median 1	median 2	KW p-value	adj. D p-value	*sign.*
1899	a	b	1475	738	1.798	1.940	<0.001	<0.001	***
1899	a	c	1475	819	1.798	1.892	<0.001	<0.001	***
1899	b	c	738	819	1.940	1.892	<0.001	0.062	
1918	a	b	394	2114	1.733	1.840	<0.001	<0.001	***
1918	a	c	394	871	1.733	1.884	<0.001	<0.001	***
1918	b	c	2114	871	1.840	1.884	<0.001	0.02	*
1933	a	b	351	2184	1.707	1.830	<0.001	<0.001	***
1933	a	c	351	1074	1.707	1.904	<0.001	<0.001	***
1933	b	c	2184	1074	1.830	1.904	<0.001	<0.001	***
1959	a	b	150	1450	1.706	1.812	<0.001	<0.001	***
1959	a	c	150	2035	1.706	1.881	<0.001	<0.001	***
1959	b	c	1450	2035	1.812	1.881	<0.001	<0.001	***
1970	a	b	138	1420	1.683	1.790	<0.001	<0.001	***
1970	a	c	138	2331	1.683	1.850	<0.001	<0.001	***
1970	b	c	1420	2331	1.790	1.850	<0.001	<0.001	***
1978	a	b	106	1473	1.701	1.800	<0.001	<0.001	***
1978	a	c	106	2509	1.701	1.860	<0.001	<0.001	***
1978	b	c	1473	2509	1.800	1.860	<0.001	<0.001	***
2020	a	b	47	493	1.830	1.791	0.024	0.856	
2020	a	c	47	3223	1.830	1.849	0.024	1.000	
2020	b	c	493	3223	1.791	1.849	0.024	0.010	*

Sign. levels: *** : p < 0.001, ** : p < 0.01, * : p < 0.05

Table S51. Kruskal–Wallis (KW) test and pairwise Dunn's (D) test with Bonferroni correction for p-values on the difference of the median fractal dimension of the settlement relationship types a, b, and c for every time step. The Dunn's test was exclusively utilized for time steps in which the p-value of the KW test was less than 0.05.

Time step	Type 1	Type 2	n 1	n 2	median 1	median 2	KW p-value	adj. D p-value	*sign.*
1899	a	b	1483	743	1.234	1.089	<0.001	<0.001	***
1899	a	c	1483	821	1.234	1.127	<0.001	<0.001	***
1899	b	c	743	821	1.089	1.127	<0.001	<0.001	***
1918	a	b	395	2123	1.310	1.163	<0.001	<0.001	***
1918	a	c	395	875	1.310	1.107	<0.001	<0.001	***
1918	b	c	2123	875	1.163	1.107	<0.001	<0.001	***
1933	a	b	351	2195	1.310	1.179	<0.001	<0.001	***
1933	a	c	351	1082	1.310	1.107	<0.001	<0.001	***
1933	b	c	2195	1082	1.179	1.107	<0.001	<0.001	***
1959	a	b	150	1458	1.321	1.200	<0.001	<0.001	***
1959	a	c	150	2044	1.321	1.121	<0.001	<0.001	***
1959	b	c	1458	2044	1.200	1.121	<0.001	<0.001	***
1970	a	b	138	1424	1.319	1.204	<0.001	<0.001	***
1970	a	c	138	2341	1.319	1.137	<0.001	<0.001	***
1970	b	c	1424	2341	1.204	1.137	<0.001	<0.001	***
1978	a	b	106	1479	1.308	1.208	<0.001	<0.001	***
1978	a	c	106	2521	1.308	1.137	<0.001	<0.001	***
1978	b	c	1479	2521	1.208	1.137	<0.001	<0.001	***
2020	a	b	47	494	1.205	1.137	<0.001	0.005	**
2020	a	c	47	3246	1.205	1.109	<0.001	<0.001	***
2020	b	c	494	3246	1.137	1.109	<0.001	<0.001	***

Sign. levels: *** : p < 0.001, ** : p < 0.01, * : p < 0.05

Reachable settlements per time step

In Fig. S10 the distribution of the number of reachable settlements is shown for different travel times and time steps. A large increase is apparent, as one would expect. The decrease in 2020 can be explained by merging of settlements around metropolitan areas, reducing the settlement count in these areas with a very high infrastructure density.

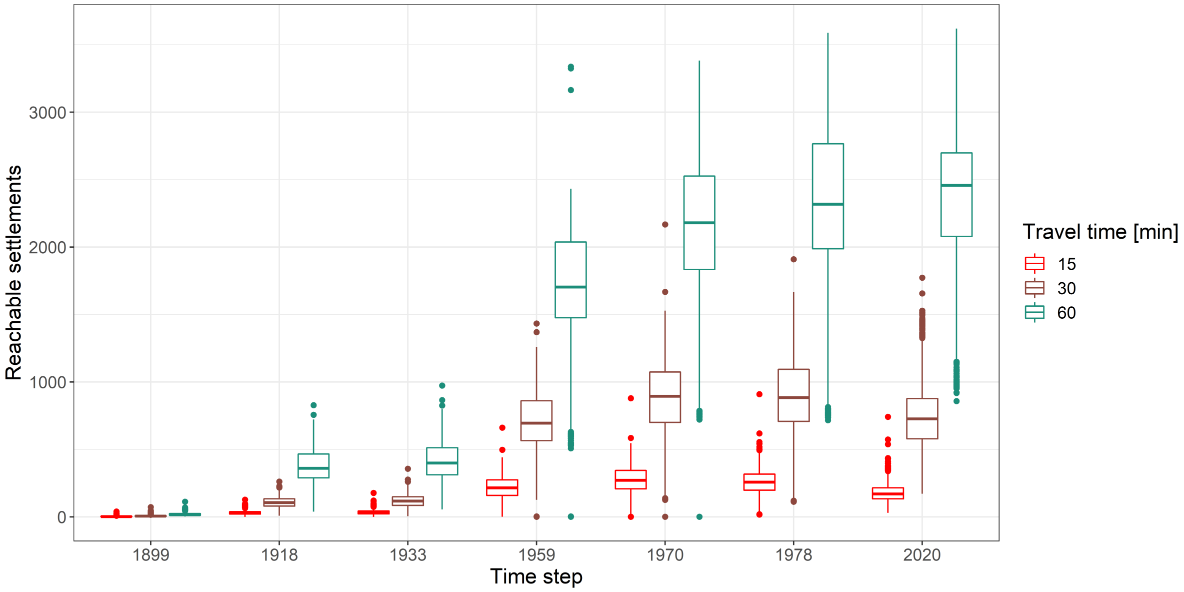

Figure S10. Distribution of reachable settlements, dependent on travel time and time step

References

1. swisstopo. Siegfried Map 1:25,000. https://www.swisstopo.admin.ch/en/geodata/maps/historical/siegfried25.html (2022). Accessed: 21/10/2022.
2. swisstopo. Old National Maps. https://www.swisstopo.admin.ch/en/geodata/maps/historical/old-national-maps.html (2022). Accessed: 21/10/2022.
3. Fröhlich, P., Frey, T., Reubi, S. & Schiedt, H. U. Entwicklung des Transitverkehrs-Systems und deren Auswirkung auf die Raumnutzung in der Schweiz (COST 340): Verkehrsnetz-Datenbank. *Arbeitsberichte Verkehrs-und Raumplanung* **208** (2005).
4. Erath, A. & Fröhlich, P. Die Geschwindigkeiten im PW-Verkehr und die Leistungsfähigkeiten von Strassen über den Zeitraum 1950-2000. *Arbeitsberichte Verkehrs-und Raumplanung* **183** (2004).
5. ARE. Nationales Personenverkehrsmodell: Zustand 2017. https://www.are.admin.ch/are/de/home/verkehr-und-infrastruktur/grundlagen-und-daten/verkehrsmodellierung/nationales-personenverkehrsmodell.html (2022). Accessed: 28/11/2022.
